# Remodeling of the gastric environment in *Helicobacter pylori*-induced atrophic gastritis

**DOI:** 10.1128/msystems.01098-23

**Published:** 2023-12-07

**Authors:** Jennifer H. B. Shuman, Aung Soe Lin, Mandy D. Westland, Kaeli N. Bryant, M. Blanca Piazuelo, Michelle L. Reyzer, Audra M. Judd, W. Hayes McDonald, Mark S. McClain, Kevin L. Schey, Holly M. S. Algood, Timothy L. Cover

**Affiliations:** 1 Department of Pathology, Microbiology and Immunology, Vanderbilt University Medical Center, Nashville, Tennessee, USA; 2 Department of Medicine, Vanderbilt University School of Medicine, Nashville, Tennessee, USA; 3 Mass Spectrometry Research Center, Vanderbilt University School of Medicine, Nashville, Tennessee, USA; 4 Department of Biochemistry, Vanderbilt University School of Medicine, Nashville, Tennessee, USA; 5 Vanderbilt Institute for Infection, Immunology, and Inflammation, Vanderbilt University Medical Center, Nashville, Tennessee, USA; 6 Veterans Affairs Tennessee Valley Healthcare System, Nashville, Tennessee, USA; Argonne National Laboratory, Lemont, Illinois, USA

**Keywords:** *Helicobacter pylori*, gastric cancer, inflammation, biomarkers, premalignant, proteomics

## Abstract

*Helicobacter pylori* colonization of the human stomach is a strong risk factor for gastric cancer. To investigate *H. pylori*-induced gastric molecular alterations, we used a Mongolian gerbil model of gastric carcinogenesis. Histologic evaluation revealed varying levels of atrophic gastritis (a premalignant condition characterized by parietal and chief cell loss) in *H. pylori*-infected animals, and transcriptional profiling revealed a loss of markers for these cell types. We then assessed the spatial distribution and relative abundance of proteins in the gastric tissues using imaging mass spectrometry and liquid chromatography with tandem mass spectrometry. We detected striking differences in the protein content of corpus and antrum tissues. Four hundred ninety-two proteins were preferentially localized to the corpus in uninfected animals. The abundance of 91 of these proteins was reduced in *H. pylori*-infected corpus tissues exhibiting atrophic gastritis compared with infected corpus tissues exhibiting non-atrophic gastritis or uninfected corpus tissues; these included numerous proteins with metabolic functions. Fifty proteins localized to the corpus in uninfected animals were diffusely delocalized throughout the stomach in infected tissues with atrophic gastritis; these included numerous proteins with roles in protein processing. The corresponding alterations were not detected in animals infected with a *H. pylori* ∆*cagT* mutant (lacking Cag type IV secretion system activity). These results indicate that *H. pylori* can cause loss of proteins normally localized to the gastric corpus as well as diffuse delocalization of corpus-specific proteins, resulting in marked changes in the normal gastric molecular partitioning into distinct corpus and antrum regions.

**IMPORTANCE:**

A normal stomach is organized into distinct regions known as the corpus and antrum, which have different functions, cell types, and gland architectures. Previous studies have primarily used histologic methods to differentiate these regions and detect *H. pylori*-induced alterations leading to stomach cancer. In this study, we investigated *H. pylori*-induced gastric molecular alterations in a Mongolian gerbil model of carcinogenesis. We report the detection of numerous proteins that are preferentially localized to the gastric corpus but not the antrum in a normal stomach. We show that stomachs with *H. pylori-*induced atrophic gastritis (a precancerous condition characterized by the loss of specialized cell types) exhibit marked changes in the abundance and localization of proteins normally localized to the gastric corpus. These results provide new insights into *H. pylori*-induced gastric molecular alterations that are associated with the development of stomach cancer.

## INTRODUCTION

*Helicobacter pylori*, a Gram-negative, spiral-shaped bacterium, persistently colonizes the stomach in about half of the human population worldwide (1). *H. pylori* colonization does not cause symptoms in most individuals, but the presence of *H. pylori* confers a markedly increased risk of peptic ulcer disease and gastric cancer (2–7). *H. pylori* colonization can trigger a chronic mucosal inflammatory response known as non-atrophic gastritis (NAG) (8–10). Some individuals subsequently develop premalignant histologic alterations, including atrophic gastritis (AG), intestinal metaplasia, and dysplasia, which can progress to gastric adenocarcinoma (2–7). Gastric cancer is a leading cause of cancer-related deaths worldwide, and infection with *H. pylori* is the strongest known risk factor for gastric cancer (11–13). Therefore, *H. pylori* has been designated as a Class I Carcinogen by the World Health Organization (14).

Progression from non-atrophic gastritis to premalignant conditions and gastric cancer is influenced by multiple factors, including strain-specific *H. pylori* genetic features, host genetic characteristics, and environmental factors (3, 4, 7, 15). One important strain-specific *H. pylori* feature contributing to carcinogenesis is the *cag* pathogenicity island (PAI), a 40-kb chromosomal locus that encodes components of the Cag type IV secretion system (Cag T4SS) and the secreted effector protein CagA (16–18). The *cag* PAI is present in some strains of *H. pylori* but not in others, and colonization with *cag* PAI-positive strains is associated with increased gastric cancer risk compared with colonization with *cag* PAI-negative strains (15). The Cag T4SS translocates CagA (designated as a “bacterial oncoprotein”), as well as LPS metabolites, DNA, and other bacterial constituents into host cells (19, 20).

The human stomach contains multiple functionally and histologically distinct regions, including the corpus and antrum (21–23). The corpus mainly functions to secrete gastric acid and contains several specialized cell lineages, including acid-secreting parietal cells and proteolytic enzyme-secreting chief cells (24). Paracrine-secreting enterochromaffin-like cells are also present in the corpus and function to stimulate gastric acid secretion by parietal cells (25). The antrum contains gastrin-secreting G cells that stimulate gastric acid secretion and somatostatin-secreting D cells that inhibit gastric acid secretion (26). Previous studies have shown that *H. pylori* preferentially colonizes the antrum at early stages of infection, leading to antral inflammation (27, 28). *H. pylori-*induced atrophic gastritis (characterized by a loss of parietal and chief cells from the corpus) can develop over the course of infection, typically beginning in the antrocorporal transitional mucosa (the junction between the antrum and the corpus) and spreading proximally along the lesser curvature of the stomach (29).

Mice and Mongolian gerbils are commonly used to investigate *H. pylori* colonization of the stomach and *H. pylori*-induced gastric alterations (30–32). Similar to human stomachs, rodent stomachs contain glandular antrum and corpus regions, as well as a non-glandular forestomach not present in human stomachs (33). Wild-type (WT) mice rarely develop severe disease in response to *H. pylori* infection, but *H. pylori*-infected Mongolian gerbils may develop gastric ulceration, premalignant conditions (atrophic gastritis and dysplasia), and gastric adenocarcinoma (31, 34–39).

Knowledge is limited regarding the molecular alterations that occur in gastric premalignant conditions such as atrophic gastritis (40, 41). In a previous study, we used a Mongolian gerbil model of gastric carcinogenesis to show that *H. pylori*-induced atrophic gastritis was accompanied by a loss of corpus-specific lipids (39). In the current study, we sought to define additional molecular alterations associated with atrophic gastritis by using transcriptional profiling, imaging mass spectrometry of tryptic peptides, and liquid chromatography with tandem mass spectrometry (LC-MS/MS) analysis of proteins in Mongolian gerbil gastric tissues. We identified 492 proteins that are preferentially localized to the gastric corpus in normal (uninfected) stomachs. We then defined two types of alterations in these corpus-specific proteins that occur in *H. pylori*-infected animals with atrophic gastritis: loss of corpus proteins (including mitochondrial proteins with key metabolic functions) and delocalization of corpus proteins involved in protein export and processing. Taken together, these data enhance our understanding of molecular alterations associated with the development of *H. pylori-*induced atrophic gastritis.

## RESULTS

### Variation in gastric inflammation severity and disease in response to *H. pylori*.

In this study, we analyzed gastric tissues from multiple cohorts of Mongolian gerbils infected for 12–16 weeks with the Cag T4SS-positive *H. pylori* strain 7.13 or mock-infected animals (Fig. S1). We also analyzed animals infected with a Δ*cagT* mutant strain, which is defective in Cag T4SS activity (Fig. S2). At the end of experiments, separate strips of each stomach were processed for histologic, transcriptomic, or proteomic analyses (further details in supplemental methods).

**Fig 1:**
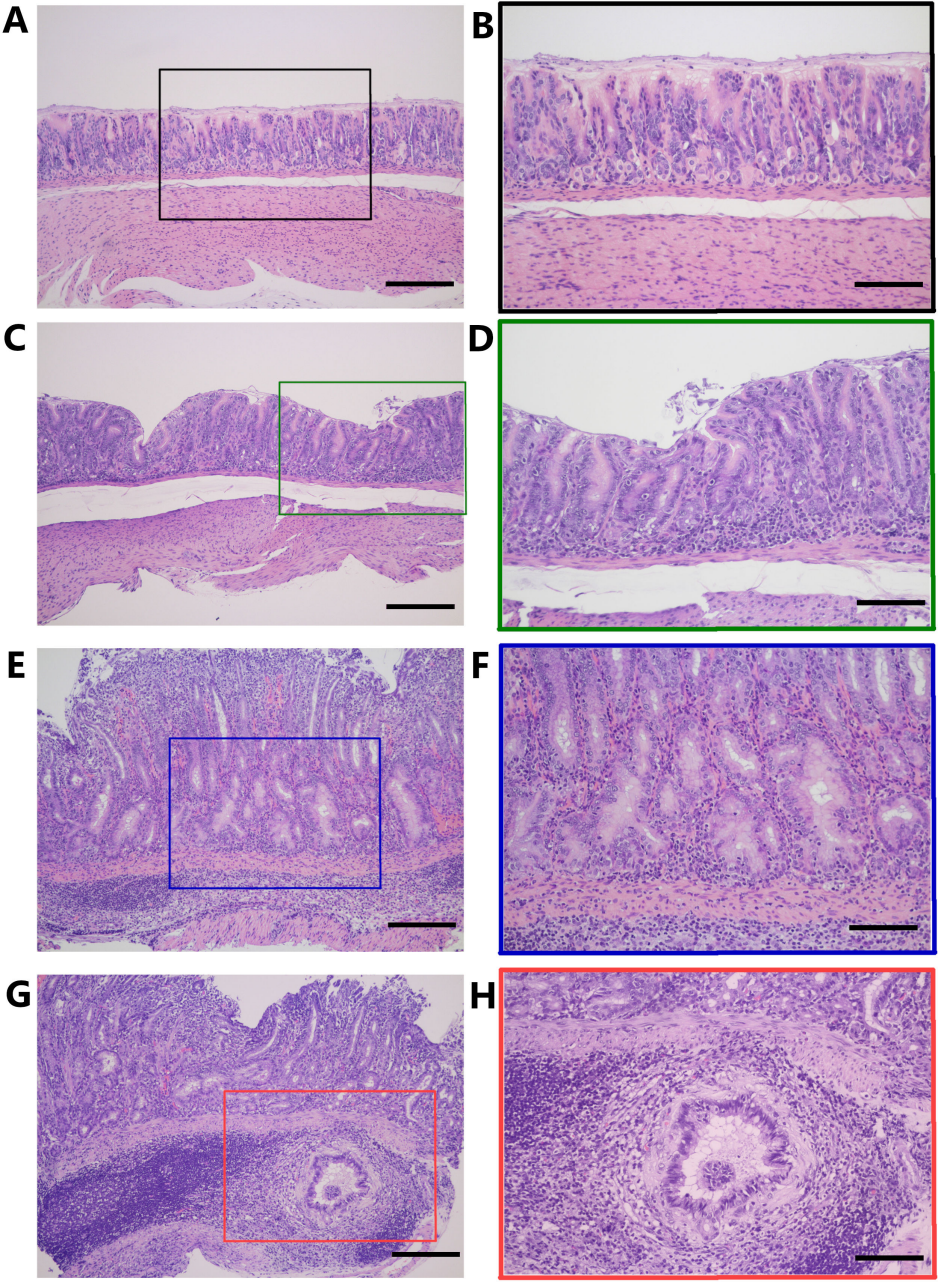
Histologic alterations in representative Mongolian gerbil gastric tissues. (**A, B**) Normal gastric histology in an uninfected animal. (**C–H**) Gastric histology in animals infected with the WT *H. pylori* strain, showing (**C, D**) mild gastritis, (**E, F**) atrophic gastritis with dysplasia, or (**G, H**) atrophic gastritis with invasive adenocarcinoma. Magnification is 100× for panels A, C, E, and G (scale bar is 200 µm) and 200× for panels B, D, F, and H, corresponding to boxed areas in panels A, C, E, and G, respectively (scale bar is 100 µm).

**Fig 2:**
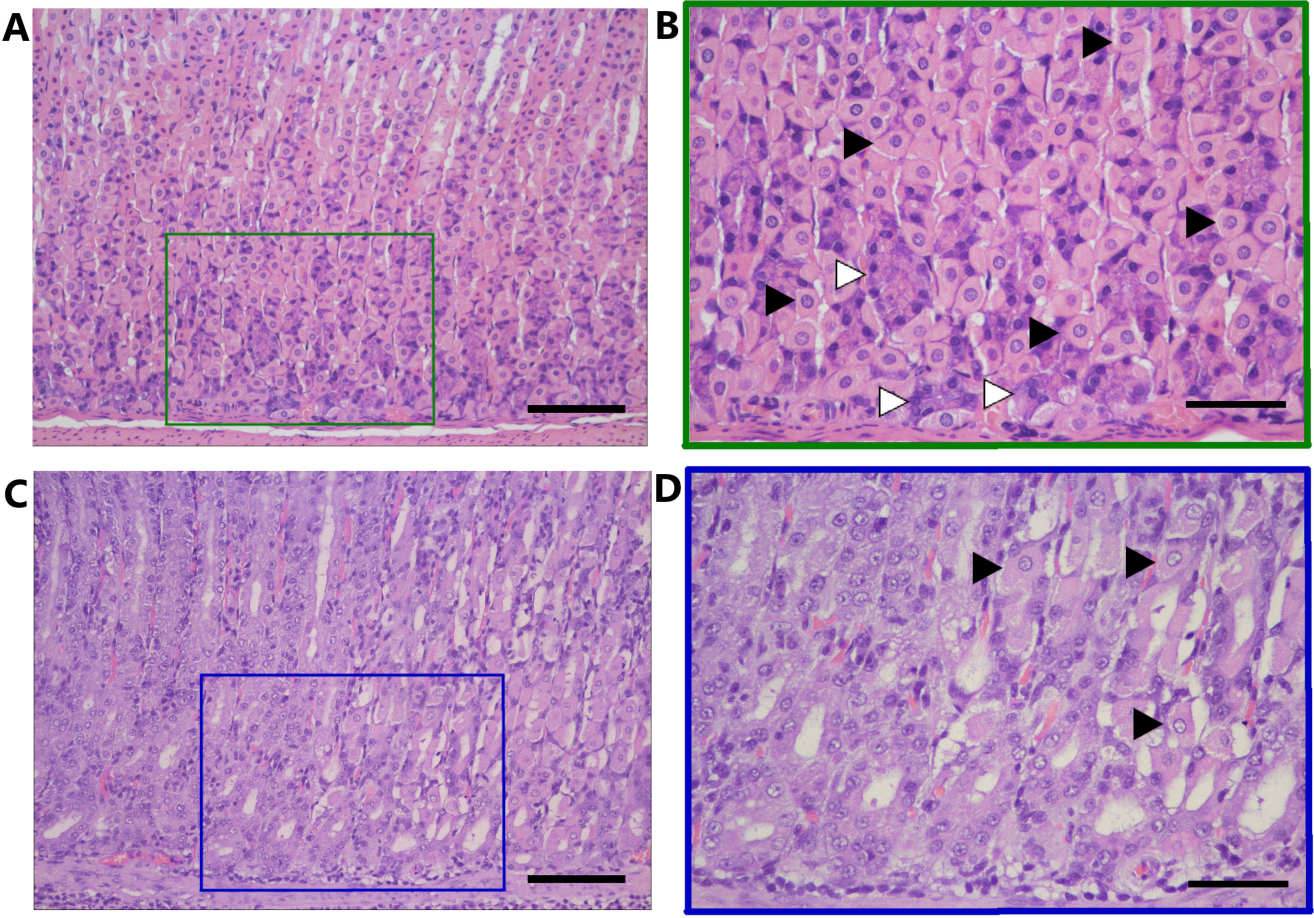
Loss of parietal and chief cells in gastric tissues from *H. pylori*-infected Mongolian gerbils exhibiting atrophic gastritis. Parietal cells (examples of which are indicated by the right-facing point of the black arrows) have a triangular or round shape and typically appear throughout corpus glands. Chief cells (examples of which are indicated by the right-facing point of the white arrows) stain a darker purple and typically appear at the base of the glands. (**A, B**) Normal corpus mucosa from an infected animal with non-atrophic gastritis, with parietal and chief cells indicated. (**C, D**) Loss of parietal and chief cells in gastric tissue from an *H. pylori*-infected animal with atrophic gastritis. Only a few viable parietal cells are observed (arrows in panel D). (**A, C**) 200× magnification (scale bar is 100 µm); (**B, D**) 400× magnification of boxed areas in panels **A** and **C**, respectively (scale bar is 50 µm).

Histopathological assessment revealed that most animals infected with WT *H. pylori* 7.13 exhibited detectable gastric inflammation, whereas none of the uninfected gerbils developed gastric inflammation (Table S1 and S2). Representative histology from these animals is shown in Fig. 1. Histologic evaluation revealed varying levels of parietal and chief cell loss (indicators of atrophic gastritis) in a subset of animals infected with the WT strain (Table S1 and 2; representative images of parietal and chief cell loss are shown in Fig. 2). Animals with non-atrophic gastritis mainly had antral inflammation, whereas animals with atrophic gastritis exhibited inflammation in both the corpus and antrum. Intestinal metaplasia was not detected in any of the gastric tissues. Dysplasia or small foci of gastric adenocarcinoma were detected in a subset of animals infected with the WT strain but not in mock-infected animals (see Fig. 1 and supplemental methods).

About 20% of animals infected with the ∆*cagT* mutant strain developed mild gastritis (scores ranging from 0.5 to 1) and the others had no detectable gastric inflammation. Atrophic gastritis, dysplasia, and invasive adenocarcinoma were not detected in any of the animals infected with the Δ*cagT* mutant strain (Table S2). Thus, experimental infection of gerbils with a WT *H. pylori* strain, but not a ∆*cagT* mutant, reproducibly resulted in gastric inflammation, atrophic gastritis, dysplasia, and gastric adenocarcinoma in a subset of animals from multiple cohorts. Consistent with the results of previous studies (36, 38), Cag T4SS activity was required for the development of premalignant lesions and gastric cancer (37).

### Transcriptional markers of key gastric cell lineages are lost in animals with atrophic gastritis

**Fig 3:**
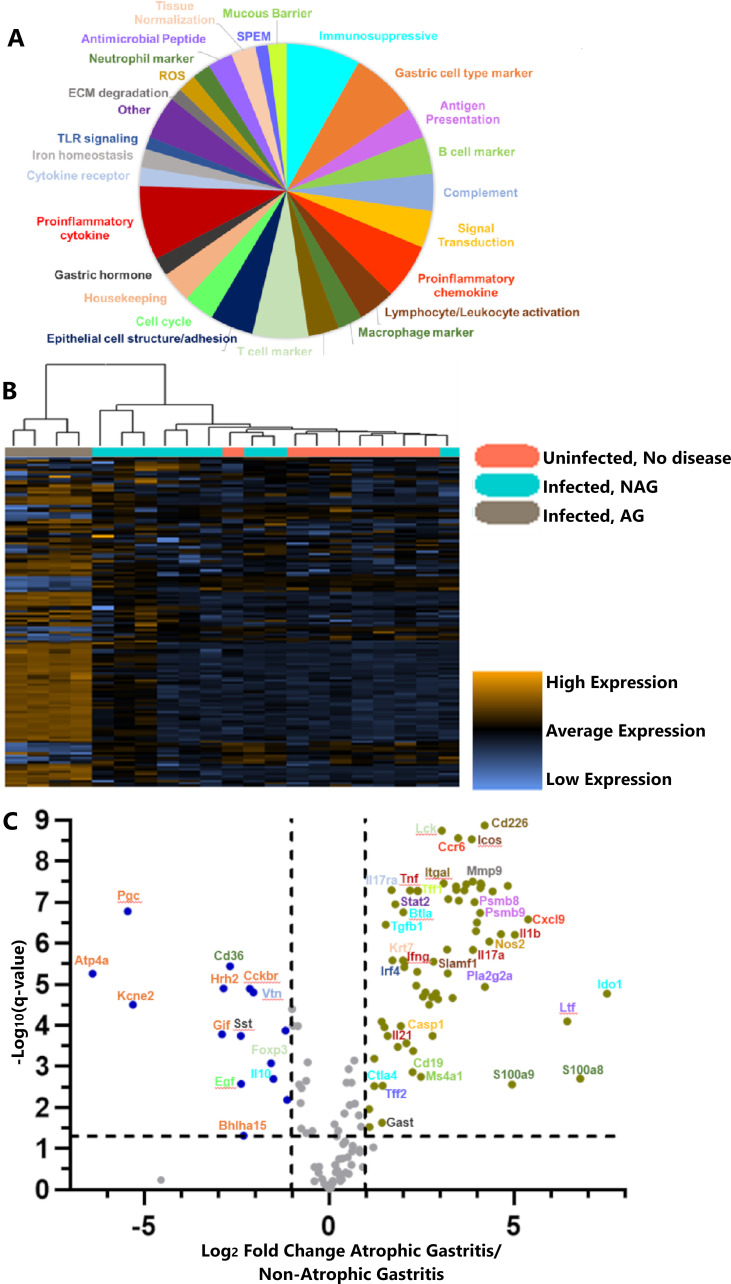
Gastric transcriptional profiles of *H. pylori*-infected and uninfected animals. Mongolian gerbils were experimentally infected with WT *H. pylori* 7.13, and gastric tissues from 12 to 16 weeks post-infection were transcriptionally profiled using a custom-designed NanoString panel. (**A**) Pie chart showing functional categories of genes represented on the custom Mongolian gerbil NanoString panel. (**B**) Agglomerative clustering (unsupervised) heat map depicting NanoString results. Each column is a different animal, and each row is a different gene. Infection status and disease state are indicated by the colored bar at the top of each column; uninfected animals are indicated by a pink bar, infected animals with non-atrophic gastritis by a teal bar, and infected animals with atrophic gastritis by a gray bar. Within the heat map, orange indicates high transcript abundance, and blue indicates low transcript abundance. (**C**) Volcano plot of differential transcript abundance in infected animals with atrophic gastritis compared with infected animals with non-atrophic gastritis. Representative genes are named and color coded based on their functional categories depicted in panel A. A more detailed analysis of differential transcript abundance is shown in Table S3. Statistical significance was evaluated using *t*-tests, followed by Benjamini-Hochberg multiple test correction.

As a first step in defining molecular alterations in tissues exhibiting atrophic gastritis, we used a custom-designed Mongolian gerbil NanoString panel containing 148 gene targets classified into 27 functional categories (described in supplemental methods and Fig. 3A) (42–44). We selected gastric tissues from a single cohort of animals for analysis (Table S1). Several tissues from each of three groups (uninfected animals with no disease, infected animals with non-atrophic gastritis, and infected animals with atrophic gastritis) were selected for analysis. In the tissues with non-atrophic gastritis, corpus inflammation was absent, the overall inflammation scores were 2.5 or less, and atrophic gastritis, dysplasia, or gastric adenocarcinoma were absent. The tissues with atrophic gastritis exhibited loss of parietal and chief cells, the presence of inflammation in the corpus, and an overall inflammation score of 8.5 or greater; all tissues with atrophic gastritis also included small foci of invasive carcinoma. RNA was isolated from stomach strips of eight uninfected animals, nine infected animals with non-atrophic gastritis, and four infected animals with atrophic gastritis. The transcriptional profiles of animals with non-atrophic gastritis were similar to those from uninfected animals and markedly different from those of infected animals with atrophic gastritis (Fig. 3B and C; Fig. S3). Of the 148 transcripts evaluated, 70 were significantly increased and 15 were significantly decreased in the animals with atrophic gastritis compared with animals with non-atrophic gastritis (Fig. 3C; Table S3). Transcriptional profiles of infected animals with atrophic gastritis compared with uninfected animals are shown in Fig. S3.

When comparing infected animals with atrophic gastritis to infected animals with non-atrophic gastritis, functional categories of genes with increased transcript counts were mostly related to immune responses; these included transcripts for proinflammatory cytokines and chemokines, antimicrobial peptides, markers of innate and adaptive immune cell activation and function, and proteins with roles in antigen presentation or generation of reactive oxygen species. Other genes with increases in transcript counts include the spasmolytic polypeptide-expressing metaplasia marker *Tff2* (45); the hormone *Gast*, elevated levels of which are associated with gastric carcinogenesis (46, 47); and the intermediate filament protein *Krt7*, increased levels of which are reported to be a negative prognostic marker in multiple cancer types (Fig. 3A and C; Table S3) (48, 49). Transcripts for several proteins involved in pathways that are known to become dysregulated in cancer, including VEGF, MAPK, and Ras pathways (represented by transcripts of *Mmp9*, *Ifgn*, *Stat2*, *Il6*, *Cdh1 Egfr*, *Tgfb1*, *Il23a*, and *Nos2*), were significantly increased in tissues with atrophic gastritis compared with infected animals with non-atrophic gastritis (Fig. 3C, Table S3) (50–52) or uninfected animals. Though some of the tissues with atrophic gastritis also contained small foci of invasive carcinoma, the relatively small proportion of tissue affected by invasive carcinoma compared with the relatively large proportion of tissue affected by atrophic gastritis suggests that the detected changes are more likely attributable to the presence of atrophic gastritis than to small foci of carcinoma.

**Fig 4:**
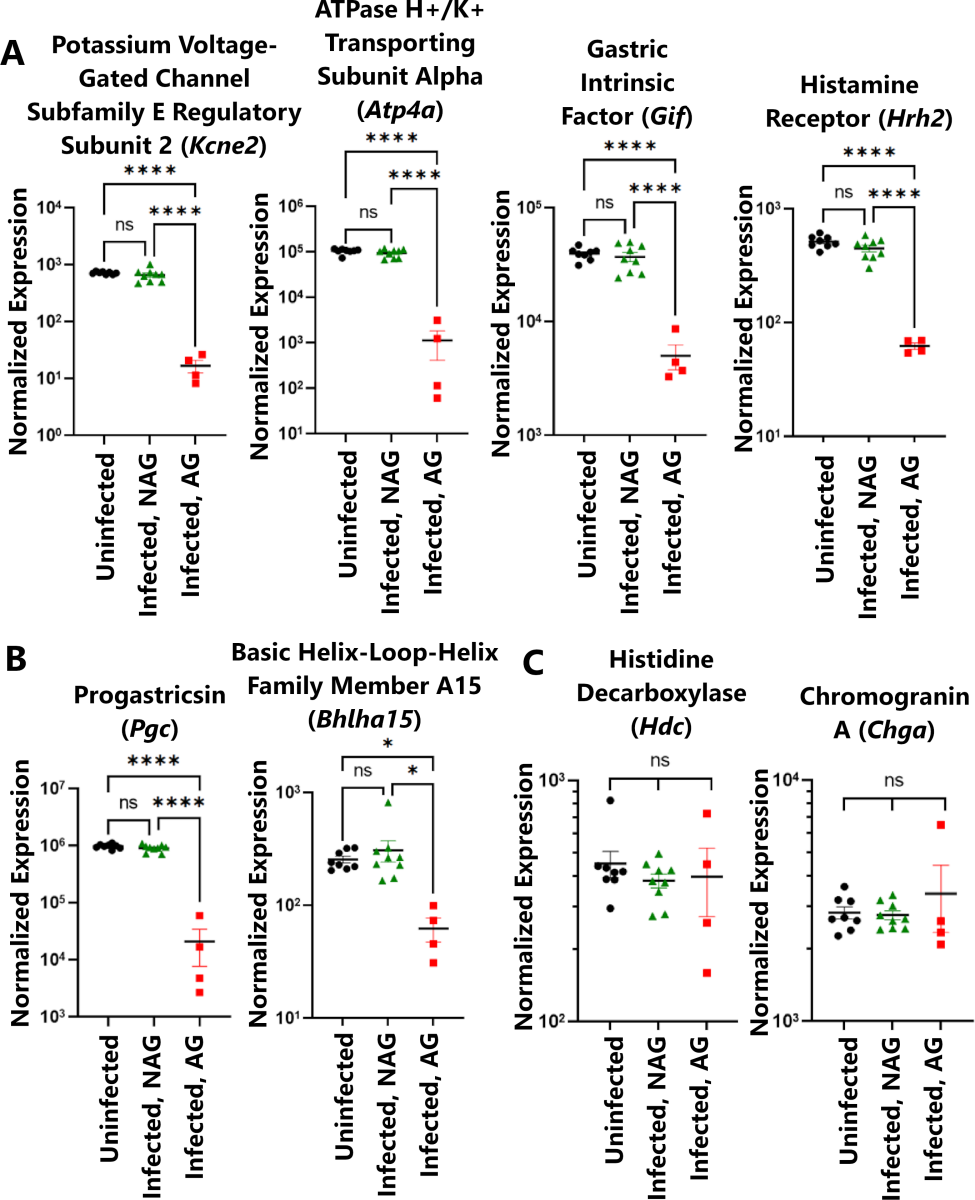
Transcript markers of specialized, corpus-specific gastric cell lineages. Transcript levels of markers for parietal cells (**A**) and chief cells (**B**) are significantly decreased in *H. pylori*-infected stomachs with atrophic gastritis compared with infected stomachs with non-atrophic gastritis or uninfected stomachs, whereas markers for enterochromaffin-like cells (**C**) show no significant difference. Three asterisks indicate *P* < 0.001; four asterisks indicate *P* < 0.0001.

Many of the genes with decreased transcript counts in the setting of atrophic gastritis were associated with specialized gastric cell types. Seven of the 15 genes with significantly decreased transcript counts were markers for cell types localized to the gastric corpus, and one (*Sst*, encoding the hormone somatostatin) is a marker for antrum-specific D cells. Transcript levels of markers for parietal cells (*Gif*, *Kcne2*, *Hrh2*, and *Atp4a*; shown in Fig. 4A) and chief cells (*Pgc* and *Bhlha15*; shown in Fig. 4B, and *Gif*) were decreased in animals with atrophic gastritis compared with infected animals with non-atrophic gastritis or uninfected animals. Transcript markers for enterochromaffin-like cells (another specialized corpus cell type marked by *Chga* and *Hdc*, shown in Fig. 3C) were not significantly changed in animals with atrophic gastritis. These findings indicate that molecular markers for parietal cells and chief cells are lost or decreased in abundance in tissues with atrophic gastritis, consistent with the histologic findings, while other specialized corpus cell types are unchanged in tissues with atrophic gastritis.

### Imaging mass spectrometry analysis of gastric tissues

As a non-targeted approach for defining alterations in tissues exhibiting atrophic gastritis, we used matrix-assisted laser desorption/ionization imaging mass spectrometry (IMS) (53, 54). IMS provides insight into the spatial distribution and relative abundance of molecules, including lipids, peptides, and proteins; in this case, gastric peptides were analyzed. In total, 22 Mongolian gerbil tissues (seven tissues from uninfected animals, three from animals infected with a Δ*cagT* mutant, four from animals infected with the WT strain and exhibiting non-atrophic gastritis, and eight from WT-infected animals exhibiting atrophic gastritis with dysplasia or invasive adenocarcinoma) were analyzed (histologic analyses presented in Table S2). Overall spectra (*m*/*z* 500 to *m*/*z* 2,000) from four representative infected stomachs with atrophic gastritis and two uninfected stomachs are illustrated and compared in Fig. S4A.

**Fig 5:**
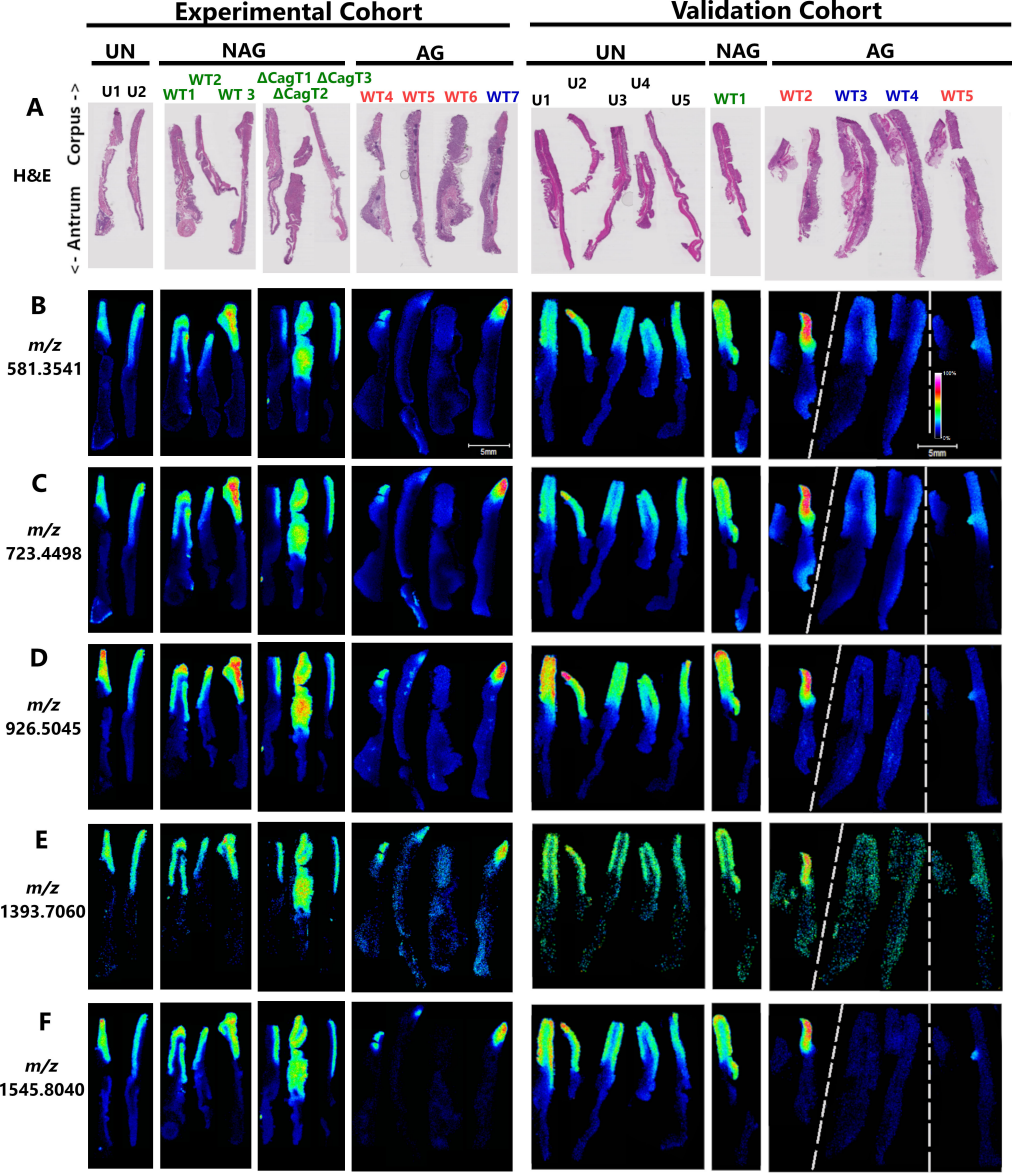
IMS analysis depicting peptides that are localized to the corpus in uninfected tissues and less abundant in *H. pylori*-infected stomachs with atrophic gastritis. Tissues from the experimental cohort are shown on the left, and tissues from the biologically independent validation cohort are shown on the right. Tissues are oriented with corpus at the top and antrum at the bottom. Infection/disease state designations are indicated above the images (UN, uninfected; NAG, non-atrophic gastritis; AG, atrophic gastritis). Individual tissues are labeled as follows: U, uninfected; WT, infected with WT *H. pylori*; Δ*cagT*, infected with the Δ*cagT* mutant strain. Black font indicates uninfected animals with normal histology, green font indicates infected animals with non-atrophic gastritis, blue font indicates infected animals with atrophic gastritis and dysplasia, and red font indicates infected animals with atrophic gastritis and small foci of invasive adenocarcinoma. The figure is a compilation of multiple tissue images joined together in a different order than that of the original slides. Scale bar: 5 mm. (**A**) H&E stains of tissues pictured in subsequent panels. (**B–F**) Ion images showing peptides *m/z* 581.3541 (ATP synthase subunit alpha, mitochondrial) (**B**), *m*/*z* 723.4498 (another match to ATP synthase subunit alpha, mitochondrial) (**C**), *m/z* 926.5045 (potassium-transporting ATPase alpha chain 1 isoform X1) (**D**), *m*/*z* 1,393.7060 (malate dehydrogenase, cytoplasmic) (**E**), and *m*/*z* 1,545.8040 (potassium-transporting ATPase subunit beta isoform X1) (**F**).

As with the transcriptional results, we detected numerous proteins that were either increased or decreased in abundance in stomachs with atrophic gastritis compared with uninfected stomachs; refer to supplemental methods for additional details. To take maximal advantage of the spatial localization afforded by IMS, we prioritized analysis of peptides that were preferentially localized to the corpus in uninfected stomachs. We compared the relative abundance and localization of these peptides in uninfected animals to their relative abundance and localization in infected stomachs with atrophic gastritis. The spectra of a representative peptide (*m*/*z* 1,545.8040, shown in Fig. 5) localized to the gastric corpus in uninfected animals and decreased in atrophic gastritis are shown in Fig. S4B. Based on independent analysis of tissues from two biological cohorts, we reproducibly identified nine peptides that were localized preferentially to the corpus in uninfected animals and decreased in abundance in tissues from most of the infected animals with atrophic gastritis (Fig. 5; these peptides are shown in a monochrome scale in Fig. S5). These corpus-specific peptides were unchanged in the corpus of infected animals with non-atrophic gastritis (infected with either the WT strain or the ∆*cagT* mutant) (Fig. 5).

**Fig 6:**
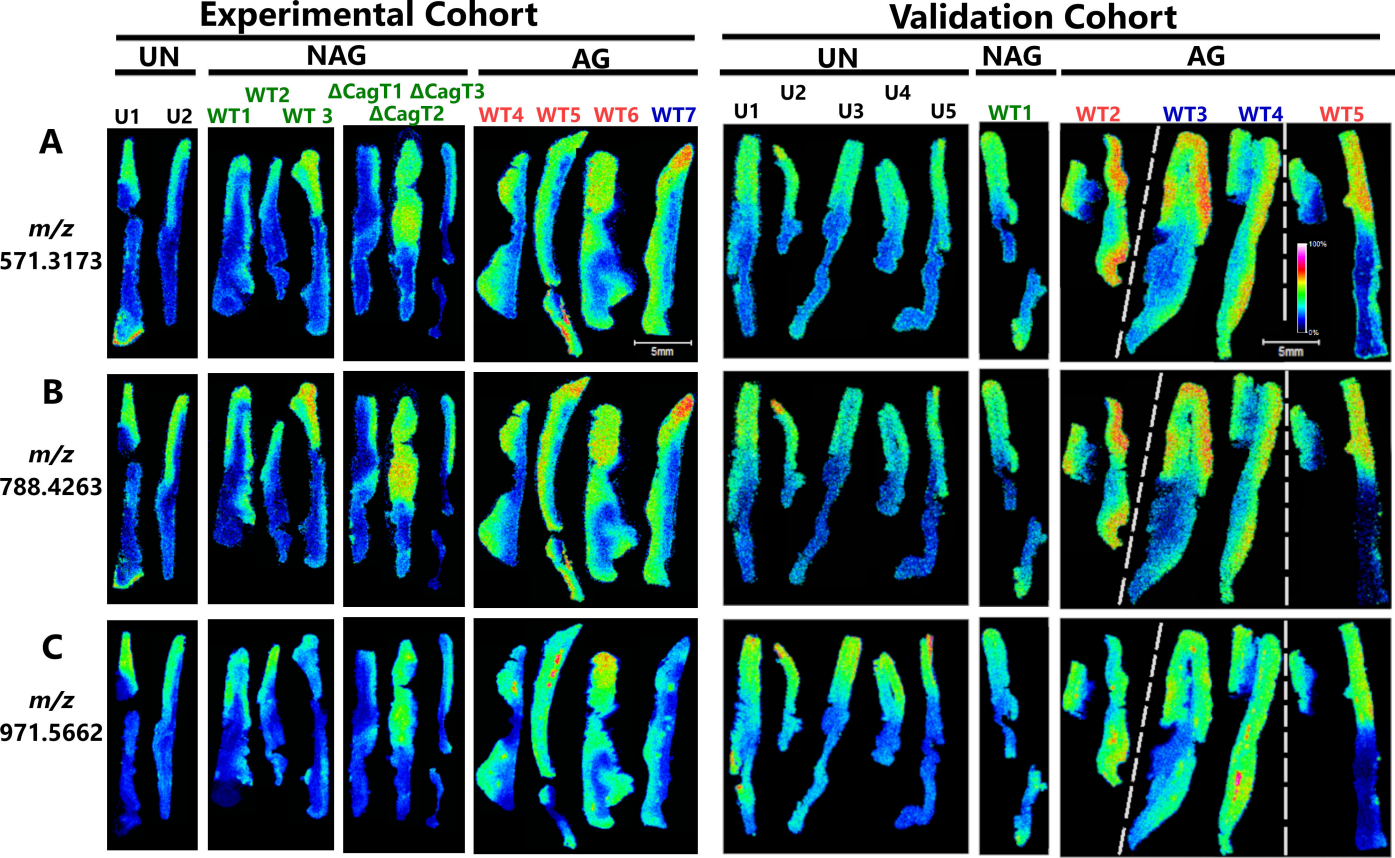
IMS analysis depicting peptides that localize preferentially to the corpus in uninfected animals and infected animals with non-atrophic gastritis but are delocalized throughout the stomach in infected animals with atrophic gastritis. Tissues from the experimental cohort are shown on the left, and tissues from the validation cohort are shown on the right. Tissues are oriented with corpus at the top and antrum at the bottom. Infection/disease state designations are indicated above the images (UN, uninfected; NAG, non-atrophic gastritis; AG, atrophic gastritis). Individual tissues are labeled as follows: U, uninfected; WT, infected with WT *H. pylori*; Δ*cagT*, infected with the Δ*cagT* mutant strain. Black font indicates uninfected animals with normal histology, green font indicates infected animals with non-atrophic gastritis, blue font indicates infected animals with atrophic gastritis and dysplasia, and red font indicates infected animals with atrophic gastritis and small foci of invasive adenocarcinoma. The figure is a compilation of multiple tissue images joined together in a different order than that of the original slides. Scale bar: 5 mm. Ions that are delocalized in atrophic gastritis include *m*/*z* 571.3173 (Filamin-B isoform X1) (**A**), *m*/*z* 788.4263 (Golgi membrane protein 1 isoform X1 or myeloperoxidase) (**B**), and *m*/*z* 971.5662 (EMILIN-1) (**C**).

Several peptides localized to the corpus in uninfected stomachs and infected stomachs with non-atrophic gastritis were not lost in tissues with atrophic gastritis but were instead diffusely delocalized throughout the stomach in most of the atrophic gastritis tissues (resulting in increased abundance in the antrum) (Fig. 6). These peptides are shown in a monochrome scale in Fig. S6. Based on the analysis of the two independent cohorts, four peptides exhibited this delocalized pattern. Notably, the four peptides that were diffusely delocalized in atrophic gastritis did not co-localize with lymphoid follicles (Fig. 5A and 6).

In total, we identified 13 peptides that were preferentially localized to the corpus in uninfected animals and exhibited reproducible delocalization or decreased abundance when comparing tissues from animals with atrophic gastritis to tissues from uninfected animals (Table 1). The infected animals with non-atrophic gastritis (including both the WT and ∆*cagT* mutant-infected animals) had gastric distributions of these 13 peptides that were more comparable to those of the uninfected animals than those of the infected animals with atrophic gastritis.

**TABLE 1:** Corpus-specific peptides lost (Fig. 5) or delocalized (Fig. 6) in tissues with atrophic gastritis, detected by IMS and identified by LC-MS/MS

Calculated peptide [M+H]^+^ ion mass (atomic mass units)	Probable IMS peptide identification(s)	NCBI reference sequence number (Mongolian gerbil)	ppm difference^*a*^	Percentage of total protein spectra	Peptide total ion current	Figure number
571.3199	Filamin-B isoform X1	XP_021502976.1	−4.551	1.18%	1.02E+8	6
581.3518	ATP synthase subunit alpha, mitochondrial	XP_021501164.1	−0.172	0.57%	1.26E+7	5
723.4513	ATP synthase subunit alpha, mitochondrial	XP_021501164.1	−2.073	0.57%	Absent	5
788.426^*b*^	Golgi membrane protein 1 isoform X1	XP_021503462.1	0.381	0.03%	8.34E+6	6
788.4336^*b*^	Myeloperoxidase	XP_021519632.1	−9.259	0.11%	4.60E+6	6
926.5054	Potassium-transporting ATPase alpha chain 1 isoform X1	XP_021498020.1	−0.971	0.32%	1.51E+8	5
958.5681	Guanylate-binding protein 5-like	XP_021517121.1	1.565	0.03%	5.11E+6	N/A
971.5634	EMILIN-1	XP_021491125.1	2.882	0.09%	6.70E+6	6
1,393.71	Malate dehydrogenase, cytoplasmic	XP_021510003.1	−2.870	0.08%	4.98E+7	5
1,545.80	Potassium-transporting ATPase subunit beta isoform X1	XP_021508721.1	8.410	0.04%	4.14E+8	5
1,619.75	Potassium-transporting ATPase alpha chain 1 isoform X1	XP_021498020.1	8.026	0.38%	1.07E+8	N/A

^*a*^ Refers to the difference between observed IMS and LC-MS/MS [M+H]^+^ ion masses (atomic mass units). The parts per million (ppm) difference calculation is described in supplemental methods.

^*b*^ The corresponding peptide detected by IMS could potentially be matched to either of these proteins.

### Identification of peptides visualized by IMS

To determine the molecular identities of the peptides of interest detected by IMS, we used reversed-phase liquid chromatography electrospray ionization tandem mass spectrometry on microextracted tryptic digest samples from adjacent tissue sections prepared at the same time, obtained following the same trypsin digestion protocol as used for IMS (additional details in supplemental methods). This analysis included gastric tissue samples from the antrum and corpus of 18 animals: 5 uninfected tissues, 3 ∆*cagT* mutant-infected tissues, 3 WT-infected tissues with non-atrophic gastritis, and 7 WT-infected tissues with atrophic gastritis. The results of LC-MS/MS analysis of the 36 samples were compared with the characteristics of the differentially abundant peptides detected by IMS, and peptide identification was performed as described in supplemental methods.

We determined putative molecular identities of seven of the nine peptides that were localized specifically to the corpus in uninfected animals but decreased in abundance in infected animals with atrophic gastritis (Table 1; Table S4; representative images shown in Fig. 5). Peptides with this pattern include *m*/*z* 926.5045 and *m*/*z* 1,619.7690 (both assigned to the potassium-transporting ATPase alpha chain, which is the catalytic subunit of the parietal cell-specific H+/K+ ATPase [55]]), *m*/*z* 1,545.8040 (potassium-transporting ATPase subunit beta, another subunit of the parietal cell specific H+/K+ ATPase; spectra for this peptide are shown in Fig. S4B), *m*/*z* 581.3541, *m*/*z* 723.4498 (ATP synthase subunit alpha, mitochondrial), and *m*/*z* 1,393.7060 (malate dehydrogenase, cytoplasmic).

In addition, we determined putative molecular identities for three of the four peptides localized to the corpus in uninfected animals but delocalized in infected animals with atrophic gastritis (Table 1; Table S4; representative images shown in Fig. 6). These peptides included *m*/*z* 571.3173 (filamin-B), *m*/*z* 788.4263 (Golgi membrane protein one or myeloperoxidase), and *m*/*z* 971.5662 (elastin microfibril interfacer 1). In total, we were able to establish probable identifications for 10 of the 13 peptides exhibiting reproducible differential abundance when comparing infected animals with atrophic gastritis to uninfected animals (Table 1; Table S4). In each case, the direction of change in peptide abundance detected by IMS matched the direction of change in protein abundance detected by LC-MS/MS.

### LC-MS/MS analysis reveals numerous alterations in gastric proteins

**Fig 7:**
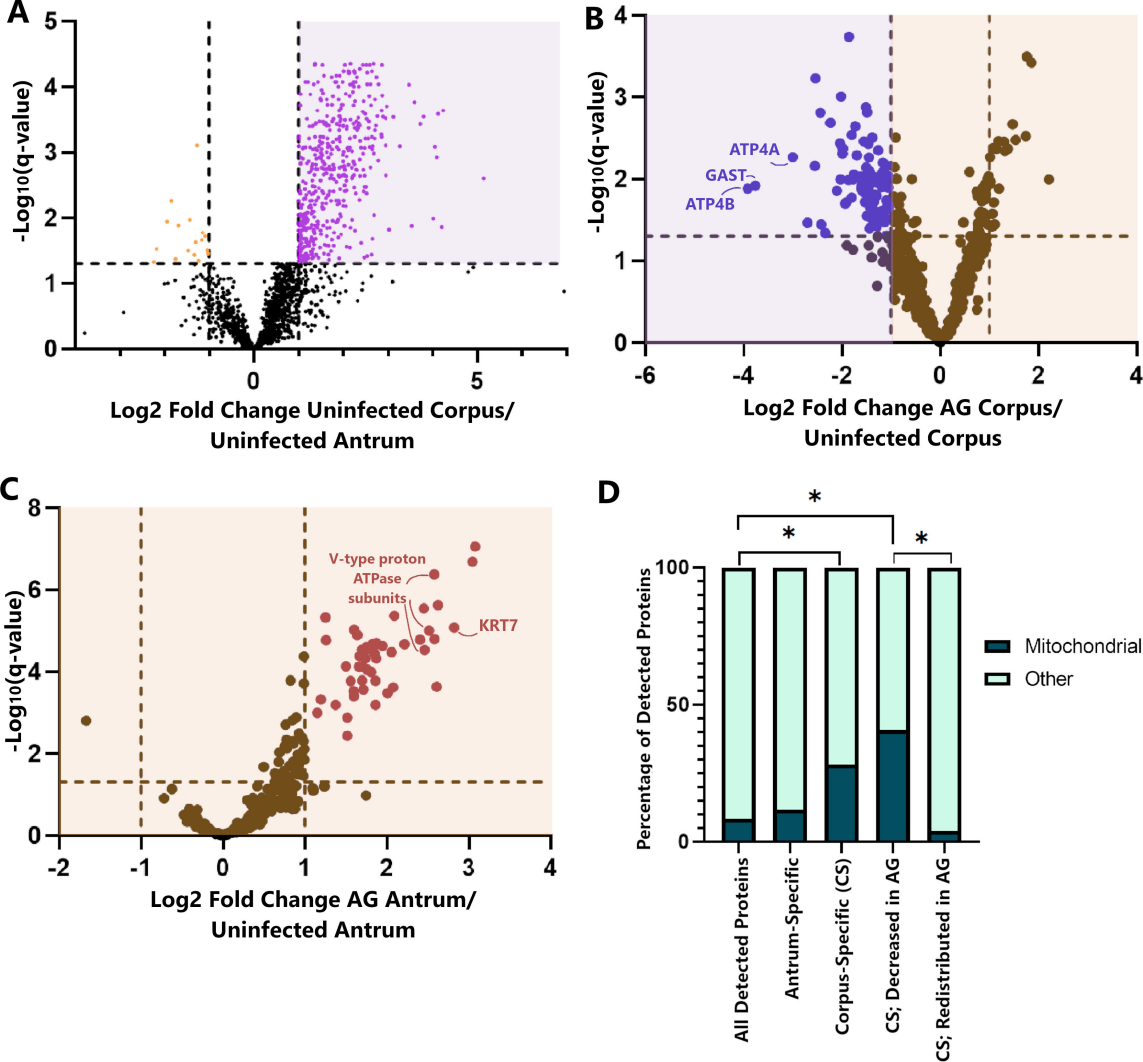
Gastric localization of proteins detected by LC-MS/MS in uninfected and infected animals. (**A**) A volcano plot comparing the abundance of proteins, determined by LC-MS/MS analysis, in the uninfected corpus compared with the uninfected antrum. Antrum-specific proteins are indicated by orange dots, and corpus-specific proteins are indicated by purple dots; proteins with specific gastric localization are listed in Table S5. (**B**) A volcano plot of the proteins shown in panel A that are corpus-specific in uninfected animals (indicated by purple shading in panel A), comparing the abundance of proteins in corpora with atrophic gastritis (AG) to that in uninfected corpora. Proteins that are decreased in atrophic gastritis are indicated by blue dots and shown in Table S6. (**C**) A volcano plot of the proteins shown in panel B that are not decreased in atrophic gastritis (indicated by yellow shading in panel B), comparing the abundance of proteins in antra with atrophic gastritis to uninfected antra. Proteins that are delocalized in atrophic gastritis are indicated by red dots and shown in Table S7. (**D**) A bar graph highlighting the increased proportion of mitochondria-annotated proteins in the subset of proteins localized to the corpus and the subset of proteins localized to the corpus that are lost in atrophic gastritis. CS, corpus-specific; AG, atrophic gastritis. * indicates *P* < 0.05.

In comparison to IMS, LC-MS/MS is a more sensitive method for protein detection, identification, and quantitation. Therefore, we used LC-MS/MS to identify additional proteins that were altered in tissues with atrophic gastritis. We did pairwise comparisons among all four treatment groups (uninfected, ∆*cagT* mutant infected, WT infected with non-atrophic gastritis, and WT infected with atrophic gastritis) and analyzed both the corpus and antrum regions from each stomach, resulting in eight total comparison groups (detailed further in supplemental methods). As with the NanoString and IMS experiments, we detected numerous proteins that were increased or decreased in abundance in infected tissues compared with uninfected tissues. We prioritized identification and analysis of proteins that were preferentially localized to either the corpus or the antrum in uninfected animals. We identified 492 proteins that were preferentially localized to the corpus and 17 proteins that were preferentially localized to the antrum in uninfected animals (Table S5; Fig. 7A).

Ninety one of the 492 proteins that localized preferentially to corpora in uninfected animals were significantly decreased in abundance in the corpora of WT-infected animals with atrophic gastritis (Table S6; Fig. 7B). Examples of corpus-specific proteins that were decreased or lost in atrophic gastritis include multiple subunits of the parietal-cell specific H+/K+ ATPase, as well as the proteolytic enzyme gastricsin (derived from the precursor progastricsin, also known as pepsinogen II, encoded by the *Pgc* gene [56]). In addition, numerous proteins involved in cellular metabolism (for example, multiple mitochondrial ATP synthase subunits, mitochondrial acetyl-CoA acetyltransferase, and multiple subunits of the cytochrome b and c complexes) were decreased in abundance in corpora with atrophic gastritis.

We observed that 50 of the 492 proteins with preferential corpus localization in uninfected animals were diffusely delocalized throughout the stomach in the setting of atrophic gastritis (Fig. 7C). Many of these proteins have essential roles in protein processing and transport, and some are known to demonstrate dysregulated expression in precancerous or cancerous tissue. For example, the delocalized protein endoplasmin, which has roles in folding proteins in the secretory pathway and is considered a key immune chaperone for the regulation of innate and adaptive immune responses (57), also contributes to cellular evasion of apoptosis (50–52). Delocalized proteins also included the intermediate filament Krt7 (which is not only delocalized but also increased in both antra and corpora in the presence of atrophic gastritis) and several components of vacuolar ATPases involved in controlling intra- and extracellular pH (58); differential expression of Krt7 and vacuolar ATPases has previously been implicated as potential negative prognostic biomarkers in several cancer types (59, 60).

Of the 3,113 gastric proteins detected by LC-MS/MS, 261 (8.4%) were annotated as mitochondrial proteins. In comparison, 141 (26.7%) of the 492 corpus-specific proteins and 2 (11.8%) of the 17 antrum-specific proteins were annotated as mitochondrial proteins (Table S5; Fig. 7D). Thus, a high proportion of the annotated mitochondrial proteins are preferentially localized to the corpus (*P* < 0.00001, Fisher’s exact test). Of the 91 proteins that were corpus-specific and decreased or lost in atrophic gastritis, 37 (40.7%) were annotated as mitochondrial (Table S6). Only 2 (4%) of the 50 proteins that were delocalized in atrophic gastritis were annotated as mitochondrial (Table S7). Thus, the set of corpus-specific proteins lost in atrophic gastritis included a higher proportion of mitochondrial proteins, compared with the set of corpus-specific proteins that were delocalized (40.7% versus 4%; *P* < 0.00001, Fisher’s exact test; Fig. 7D).

**Fig 8:**
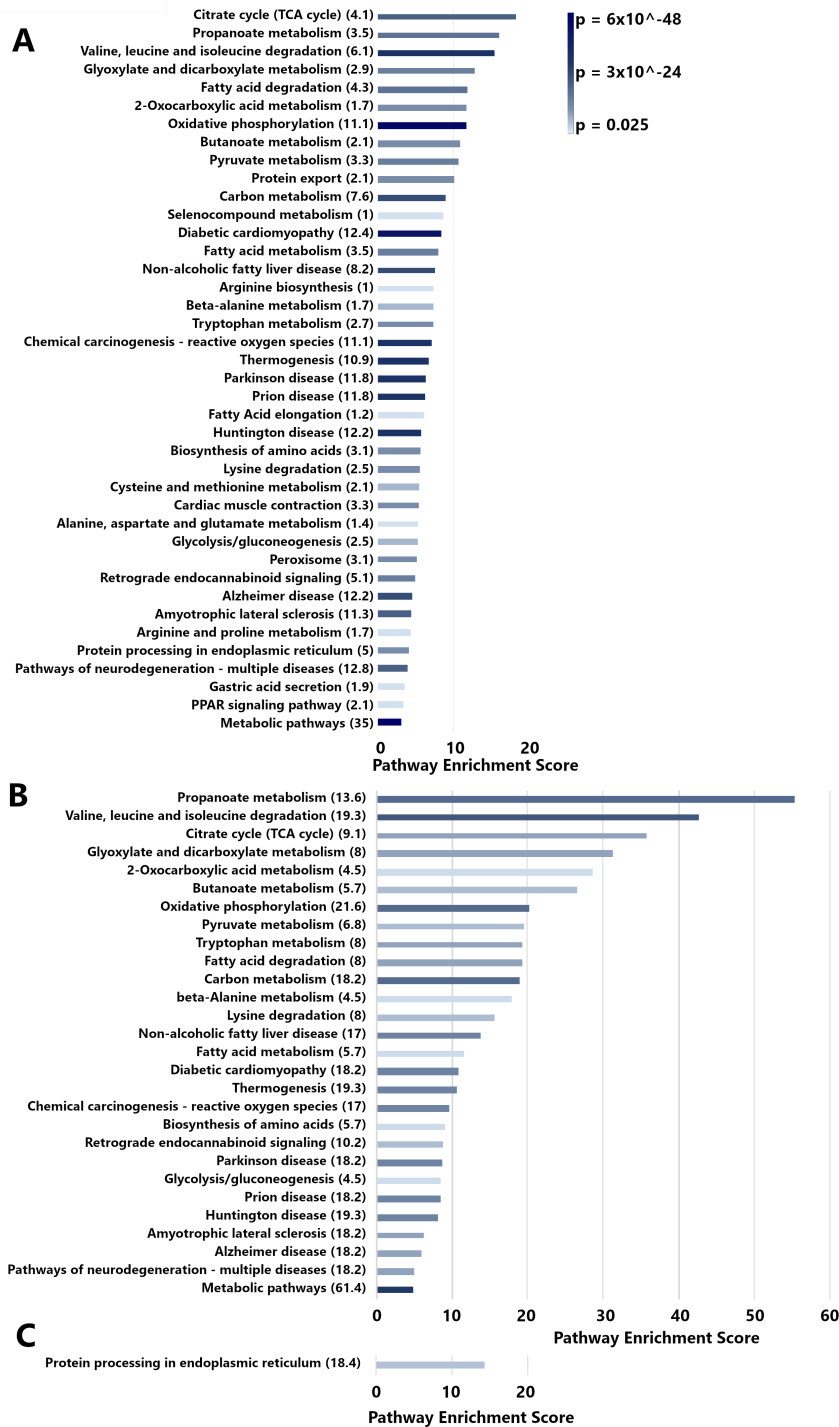
Pathway analysis of corpus-specific proteins altered in atrophic gastritis. Corpus-specific proteins identified by LC-MS/MS (shown in Table S5 to S7) were analyzed as described in supplemental methods, using DAVID. Pathways are denoted at the left. Parentheses indicate the percentage of genes annotated in the pathway compared with the number of genes analyzed (also known as gene ratios). The pathway enrichment score (defined in supplemental methods) is plotted along the *x*-axis, and the bar is colored according to its *P*-value (determined by an EASE score, which is calculated using a modified Fisher’s exact test; more details in supplemental methods). Only pathways with statistically significant enrichment are shown. (**A**) Pathway analysis of genes encoding proteins that are localized preferentially to the corpus in uninfected animals. (**B**) Pathway analysis of genes encoding proteins that are corpus-specific in uninfected animals and decreased in abundance in atrophic gastritis. (**C**) Pathway analysis of genes whose proteins are corpus-specific in uninfected animals and delocalized diffusely throughout the stomach in atrophic gastritis.

As a next step in analysis, we used the Database for Annotation, Visualization, and Integrated Discovery (DAVID) to classify proteins with altered abundance into KEGG pathways (61). Proteins localized to the corpus in uninfected stomachs were classified into about two dozen pathways related to metabolism, multiple pathways related to cellular signaling or protein generation, and gastric acid secretion (Fig. 8A). The subset of corpus-specific proteins with reduced abundance in atrophic gastritis was mostly classified into pathways related to metabolism of amino acids, short-chain fatty acids, and other molecules (Fig. 8B; annotation of genes to pathways is shown in Table S9). About 63% of the proteins that mapped to the “metabolic pathways” KEGG pathway were annotated as mitochondrial proteins. The subset of corpus-specific proteins that were delocalized in atrophic gastritis were classified into the “protein processing in the endoplasmic reticulum” pathway (Fig. 8C). Notably, there was no overlap in the pathways for proteins lost in atrophic gastritis compared with pathways for delocalized proteins.

To evaluate whether the gastric protein alterations were associated with *H. pylori-*induced atrophic gastritis or with infection alone, proteomic profiles from the corpora and antra of uninfected animals were compared with those of animals with non-atrophic gastritis (infected with the ∆*cagT* mutant or WT strain). The proteomic profiles of infected animals with non-atrophic gastritis were remarkably similar to those from uninfected animals (Table S8). Several alterations were detected in both infected animals with non-atrophic gastritis and infected animals with atrophic gastritis when each was compared with uninfected animals (marked with a pound sign in Table S8). Only 10 changes in protein abundance were detected in all groups of infected animals (infected with either the WT strain or the ∆*cagT* mutant and all disease states) but not in uninfected animals; these infection-dependent changes are marked with an asterisk in Table S8. Overall, these findings indicate that *H. pylori* can cause gastric molecular alterations in the absence of atrophic gastritis, but remodeling of the gastric environment is far more extensive in cases of *H. pylori*-induced atrophic gastritis.

## DISCUSSION

*H. pylori* colonization of the human stomach triggers a mucosal inflammatory response and cellular alterations that can ultimately result in gastric adenocarcinoma. Similar to what is observed in humans, *H. pylori*-infected Mongolian gerbils can develop gastric mucosal inflammation, premalignant lesions (including atrophic gastritis), and gastric cancer. In this study, we detected two distinct remodeling processes involving proteins that are preferentially localized to the gastric corpus in normal stomachs and lost or delocalized in atrophic gastritis.

Both loss and delocalization of corpus-specific proteins were detected in animals infected with the WT *H. pylori* strain but not in animals infected with a ∆*cagT* mutant strain. Previous studies have shown that CagT is a component of the Cag T4SS outer membrane core complex, and Δ*cagT* mutants exhibit loss of Cag T4SS function (38, 62–67). A previous study, utilizing a strain in which *cagT* was controlled by a TetR/*tetO*-inducible expression system, showed that the *cagUT* operon is required for *H. pylori*-induced gastric carcinogenesis in a Mongolian gerbil model (38). CagA is the only protein known to be secreted by the Cag T4SS. Previous studies have shown that the heterologous expression of CagA in transgenic mouse, zebrafish, or *Drosophila* models is sufficient to cause hyperproliferation of gastric cells and gastric cancer (68–72). The observed dependence of gastric remodeling on Cag T4SS activity in the current study is consistent with results of other studies indicating that the Cag T4SS and the oncoprotein CagA cause cellular alterations relevant to cancer pathogenesis (35, 38, 73).

We detected the loss of several parietal cell markers in *H. pylori*-induced atrophic gastritis, using both targeted transcriptional profiling and untargeted proteomic approaches. For example, levels of the parietal cell-specific H+/K+ ATPase and the chief cell-specific enzyme gastricsin (or progastricsin) were decreased at both the transcriptional level (loss of *Atp4a* and *Pgc*) and at the proteomic level (loss of the alpha and beta ATPase subunits and loss of gastricsin). The untargeted proteomic analyses allowed us to detect *H. pylori*-induced loss of additional corpus-specific proteins not previously known to be altered in atrophic gastritis. The current study was not designed to allow a comprehensive comparison of transcriptional and proteomic results, and many of the transcripts analyzed with the NanoString panel were not detectable at a protein level using untargeted proteomic approaches. Nevertheless, the data from the transcriptional and proteomic studies were generally in agreement; there were no examples of markers for corpus-specific cell types that changed in one way at the transcriptional level and changed in the opposite way at the proteomic level.

In the normal stomach, parietal and chief cells are localized to the corpus but not the antrum, and somatostatin-producing D cells are localized in the antrum but not the corpus (22, 23). Previous work has shown that continuous replenishing of these region-specific cell types depends on distinct populations of stem cells and transcription factors for each compartment (21, 23, 74, 75). Most previous studies have used histologic methods to differentiate the gastric corpus and antrum regions. In the current study, we used two proteomic methods to assess protein localization in the stomach (IMS and comparative LC-MS/MS analyses of corpus and antrum samples). Each method used the same trypsin digestion protocols, which facilitated a comparison of the two sets of data and identification of peptides detected by IMS. Using LC-MS/MS, we identified 492 proteins that are preferentially localized to the corpus and 17 proteins preferentially localized to the antrum in uninfected stomachs (Fig. 7; Table S5). The relatively large number of proteins preferentially localized to the corpus probably reflects the specialized functions and cell types of the corpus.

A previous study of human gastric tissue reported the detection of 436 corpus-specific proteins in histologically normal human tissues (76). Among the 492 corpus-enriched proteins detected in uninfected Mongolian gerbil tissues in the current study, 117 were also reported to be corpus-enriched in normal human gastric tissues; an overlap of these proteins is shown in Fig. S7, and the unique and shared proteins are listed in Table S10. Shared proteins included the parietal cell-specific H+/K+ ATPase subunits ATP4A and ATP4B as well as many mitochondrial proteins. A pathway analysis of the corpus-specific proteins detected in both normal human and uninfected Mongolian gerbil gastric tissues revealed results similar to the results from pathway analysis of corpus-specific proteins detected in the Mongolian gerbil tissues, with the greatest enrichment in the “metabolic pathways” KEGG pathway. Fifty-one of the 117 shared corpus-specific proteins (43.5%) were decreased in abundance in gastric tissues from human patients with gastric cancer.

Almost one-third of the 492 proteins that were corpus-specific in normal (uninfected) gerbil stomachs were decreased in abundance or delocalized diffusely throughout the stomach in animals with atrophic gastritis. Altered abundance or altered localization of most of these proteins has not been previously described. The loss of corpus-specific proteins detected in *H. pylori*-infected Mongolian gerbils with atrophic gastritis parallels the loss of corpus-specific lipids detected in a previous study (39).

Pathway analysis showed that a high proportion of the corpus proteins lost in atrophic gastritis have metabolic functions (Fig. 7D and 8B). These include numerous proteins with functions in the mitochondrial electron transport chain, redox balance, beta oxidation of fatty acids, and branched chain amino acid synthesis and degradation. About two-thirds of the proteins that mapped to the “metabolic pathways” KEGG pathway were annotated as mitochondrial proteins. The detection of alterations in mitochondrial proteins is consistent with the results of previous studies (*in vitro*, using animal models, or using human gastric tissue), which reported that *H. pylori* infection can lead to dysregulation of mitochondrial function (77, 78). These results indicate that *H. pylori* causes alterations in both mitochondrial proteins and non-mitochondrial proteins with metabolic functions. Notably, we detected these types of protein alterations only in the gastric corpus and not in the antrum.

Gastric acid secretion requires substantial energy, and accordingly, parietal cells contain very high numbers of mitochondria (79, 80). As such, we hypothesize that the abundance of mitochondrial proteins in uninfected corpora (and loss of mitochondrial proteins from the corpus in atrophic gastritis) reflects the presence or loss of mitochondria-rich, corpus-specific parietal cells. To further explore this hypothesis, we analyzed several proteins previously reported to be parietal cell tubulovesicle compartment constituents that exhibited corpus-specific localization in uninfected animals in the current study (Rab11, syntaxin 12, and vesicle-associated membrane protein 8; Table S5) (81). Levels of these tubulovesicle proteins were somewhat lower in corpora with atrophic gastritis compared with uninfected corpora, but the differences were not statistically significant. Since the loss of mitochondrial proteins in atrophic gastritis was more prominent than the loss of parietal cell tubulovesicle compartment components, it is unlikely that the decreased abundance of mitochondrial proteins in atrophic gastritis is solely due to the loss of parietal cells.

In addition to detecting the loss of specific proteins normally localized to the corpus, we unexpectedly detected diffuse delocalization of proteins normally localized to the gastric corpus (Table S7). This delocalization was characterized by an increase in abundance of proteins in the antrum, with or without a corresponding increase in abundance in the corpus. Importantly, the functional properties of the delocalized proteins are different from those of the proteins that were reduced in abundance. Most of the delocalized proteins have roles related to protein processing. Specifically, delocalized proteins have roles in altered cellular processes during stress (e.g., hypoxia upregulated protein 1); protein folding and unfolding (e.g., UDP-glucose:glycoprotein glucosyltransferase 1); and protein degradation (e.g., endoplasmin). Delocalized proteins are involved in processes that occur in multiple cellular locations, including transport through the Golgi network and multiple types of vesicles (e.g., multiple different coatomer subunits); protein trafficking between the Golgi complex and endoplasmic reticulum (e.g., ADP-ribosylation factor GTPase-activating proteins 2 and 3); and endoplasmic reticular proteins (e.g., cytoskeleton-associated protein 4). Interestingly, the aberrant or increased expression of several delocalized proteins (hypoxia upregulated protein 1, protein LYRIC, keratin type II cytoskeletal 7 [Krt7], and sodium/potassium-transporting ATPase subunit alpha-1) has been associated with carcinogenesis (48, 82–85).

*Krt7* (one of the proteins delocalized in atrophic gastritis) encodes an intermediate filament protein expressed in simple glandular epithelia and transitional epithelia, blood vessels, and gland ducts in the epithelial cells of some internal organs (lungs and breast tissue) but not others (colon and prostate tissue) (86). High expression of *Krt7* in carcinoma has been reported as an independent factor for poor prognosis of multiple types of cancer, including breast, colorectal, prostate, and lung cancers (48, 49). In normal stomach tissue, *Krt7* expression is very low compared with its expression in other organs, and increased levels of *Krt7* have recently been implicated as a potential biomarker of gastric carcinogenesis (59, 87, 88). In the current study, Krt7 was also increased at the transcriptional level in cases of atrophic gastritis.

The delocalized proteins did not co-localize with lymphoid follicles in *H. pylori*-infected animals, and we did not detect evidence of gastric inflammation in uninfected animals. Therefore, the delocalization of corpus-specific proteins detected in the current study is probably not solely attributable to the development of an *H. pylori*-induced gastric mucosal inflammatory response. Instead, it is more likely that these proteins become diffusely delocalized throughout the stomach in response to *H. pylori*-induced changes in the normal regulatory processes that maintain differences in the cellular content, glandular structure, and protein content of the corpus compared with the antrum.

The term “antralization” has been used to describe the loss of corpus-defining cell lineages in association with *H. pylori-*induced mucosal inflammation, resulting in the replacement of the specialized corpus cell lineages with mucous cells and transformation of corporal glands into more antral-like glands (89–94). Previous studies of atrophic gastritis have emphasized the loss of specialized parietal and chief cells in the corpus, along with high levels of inflammation. The results of the current study indicate that the *H. pylori*-induced changes in gastric organization are more complex than previously recognized. Instead of a simple antralization transition (converting corpus mucosa to antral mucosa), these results indicate that there is a concomitant delocalization of proteins normally found in the gastric corpus into the gastric antrum. Overall, the gastric remodeling associated with *H. pylori*-induced disease may be more accurately described as a blurring of the normal boundaries between the gastric corpus and the antrum, instead of the spread of antrum-like mucosa throughout the stomach.

In summary, this study provides important new insights into the molecular alterations that occur in *H. pylori*-induced atrophic gastritis. Collectively, the results indicate that *H. pylori* colonization can cause a disruption of the normal organization of the stomach into histologically and functionally distinct corpus and antrum regions; this loss of functional and structural distinction likely contributes to gastric cancer pathogenesis.

## MATERIALS AND METHODS

### *H. pylori* culture methods

For descriptions of *H. pylori* culture methods and generation of the Δ*cagT* mutant strain, please refer to supplemental methods.

### Mongolian gerbil infections

Mongolian gerbils were infected with *H. pylori* strain 7.13 or an isogenic ∆*cagT* mutant strain, as described in supplemental methods and Fig. S1. Animals were euthanized at 12 or 16 weeks post-infection. Gastric tissue was processed for culture, histology, and other analyses as described in supplemental methods.

### RNA extraction and transcriptional analysis

RNA was extracted from whole strips of stomach tissue using the acid-guanidinium-phenol (TRIzol)-chloroform method and analyzed using a custom-designed Mongolian gerbil NanoString panel. Results were analyzed using nSolver and Advanced Analysis software. Additional details are in supplemental methods.

### MALDI mass spectrometry sample preparation and analysis

Fresh frozen stomach tissues were sectioned into 12-µm thick sections using a cryostat (Leica CM 3050S, Leica Biosystems, Nußloch, Germany). Samples were washed and digested overnight as previously described (95). A 15 Tesla Bruker SolariX FT-ICR mass spectrometer was used to acquire tryptic peptide images. We used flexImaging software (Bruker Daltonics) to manually analyze each peak in the mass spectrum and thereby identify signals of interest; no automated algorithms or supplemental analysis programs were used. We first analyzed the data to detect imaging peaks that were enriched in the corpus (compared with the antrum) in tissues from uninfected animals. We then did further analysis of these corpus-specific peaks to identify those that were decreased in abundance or delocalized in tissues from *H. pylori*-infected animals with atrophic gastritis compared with tissues from uninfected animals. Additional details are in supplemental methods.

### LC-MS/MS sample preparation and analysis

Antrum and corpus regions of the stomach were delineated by histologic analysis and analyzed separately by LC-MS/MS. Following trypsin digestion of specific regions of the tissue sections overnight, peptides were microextracted and dried. Samples were analyzed via reversed phase column coupled to a nano-electrospray source with an Exploris 480 (Thermo-Fisher) mass spectrometer. Peptides were resolved using an RSLC-nano (Thermo-Fisher). LC-MS/MS data were analyzed and identified using FragPipe (software can be found at nesvilab.org) and IonQuant (96). Results were searched against a Mongolian gerbil protein database appended to an *H. pylori* protein database, with default parameters for LFQ-MBR searches performed using MSFragger (97). Search results filtered to a 1% false discovery rate using Percolator (98) were loaded into Scaffold (Proteome Software Inc., Portland, OR) for visualization. Pairwise comparisons between groups and gastric regions were performed using MSStats (99). Additional details, including information about pathway analysis, are in supplemental methods.

### Matching IMS images to LC-MS/MS peptide identifications

To compare LC-MS/MS data to IMS data with the goal of matching probable identifications for imaged peptides of interest, we calculated a parts per million (ppm) difference (Table 1; Table S4). Additional details are in supplemental methods.

### Pathway analysis

Accession numbers of Mongolian gerbil proteins of interest identified in the LC-MS/MS analysis were converted to mouse gene names to facilitate pathway analysis. Official gene symbols were entered into DAVID. The resulting KEGG pathways were used for further enrichment analysis. Additional details are in supplemental methods.

## Data Availability

The mass spectrometry proteomics data have been deposited to the ProteomeXchange Consortium via the PRIDE partner repository with the data set identifier PXD046333.

## SUPPLEMENTAL MATERIAL

Fig. S1 msystems.01098-23-s0001.tif Description of Mongolian gerbil cohorts.

Supplemental figure legends msystems.01098-23-s0002.pdf Legends for Figures S1 to S7.

Fig. S2 msystems.01098-23-s0003.tif Loss of Cag T4SS activity in *cagT* mutant strains.

Fig. S3 msystems.01098-23-s0004.tif Gastric transcriptomes of *H. pylori*-infected animals with atrophic gastritis compared to uninfected animals.

Fig. S4 msystems.01098-23-s0005.tif Averaged spectra of gastric peptides from representative gerbil stomachs.

Fig. S5 msystems.01098-23-s0006.tif IMS analysis depicting peptides localized to the corpus in uninfected tissues and decreased in abundance in infected stomachs with atrophic gastritis (monochrome images).

Fig. S6 msystems.01098-23-s0007.tif IMS analysis depicting peptides localized preferentially to the corpus in uninfected animals and delocalized throughout the stomach in infected animals with atrophic gastritis (monochrome images).

Fig. S7 msystems.01098-23-s0008.tif Cross-species comparison of corpus-enriched proteins.

Additional experimental details msystems.01098-23-s0009.pdf Supplemental methods.

Supplemental tables msystems.01098-23-s0010.xlsx Tables S1 to S10.

ASM does not own the copyrights to Supplemental Material that may be linked to, or accessed through, an article. The authors have granted ASM a non-exclusive, world-wide license to publish the Supplemental Material files. Please contact the corresponding author directly for reuse.
