# Supplementary figures and images for: Remodeling of the gastric environment in Helicobacter pylori-induced atrophic gastritis
Source: mSystems. 2023 Dec 7;9(1):e01098-23. doi: 10.1128/msystems.01098-23 (PMC10805037; doi:10.1128/msystems.01098-23)

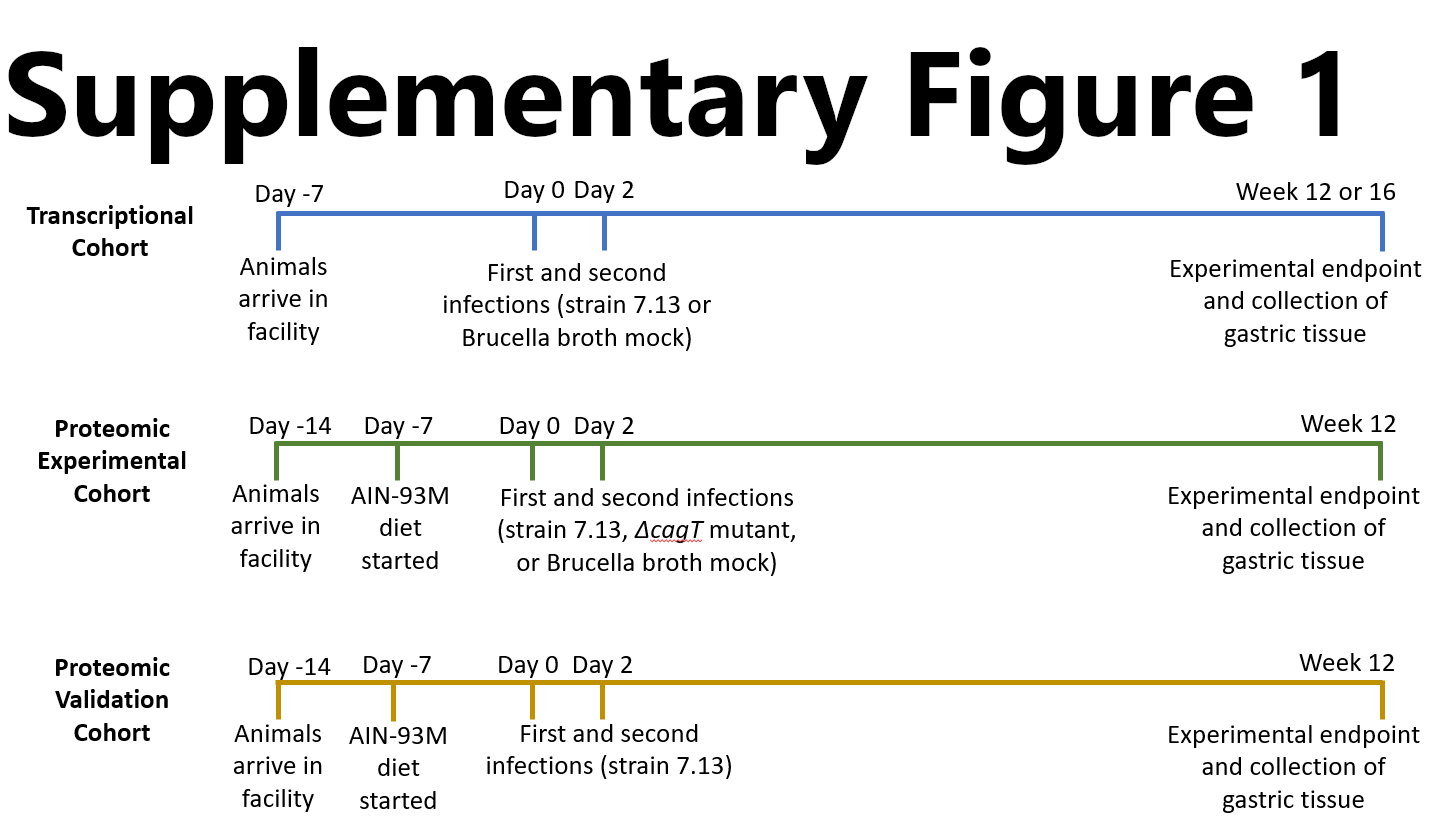

Supplement: Fig. S1 — Description of Mongolian gerbil cohorts. [file msystems.01098-23-s0001.tif]

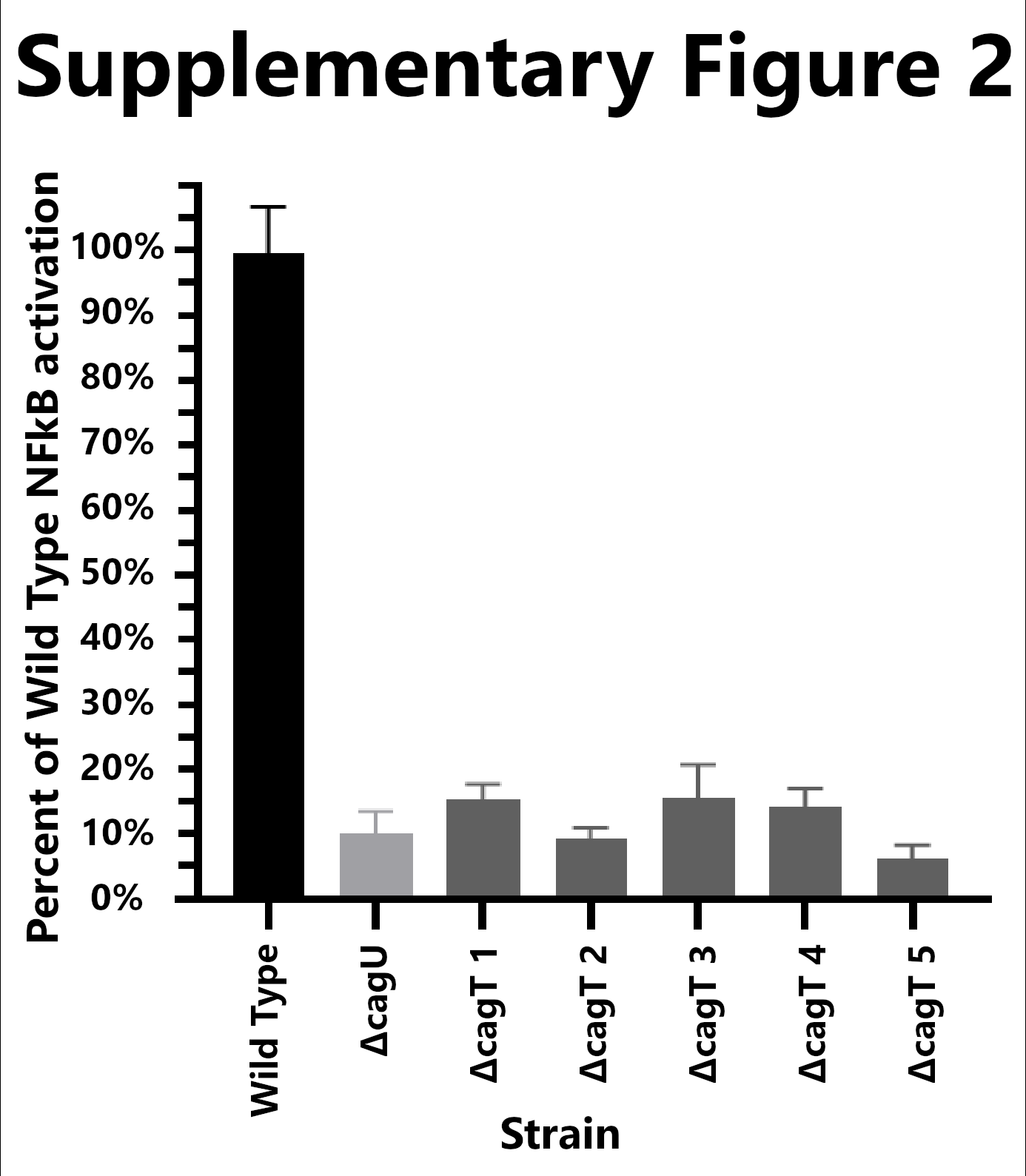

Supplement: Fig. S2 — Loss of Cag T4SS activity in cagT mutant strains. [file msystems.01098-23-s0003.tif]

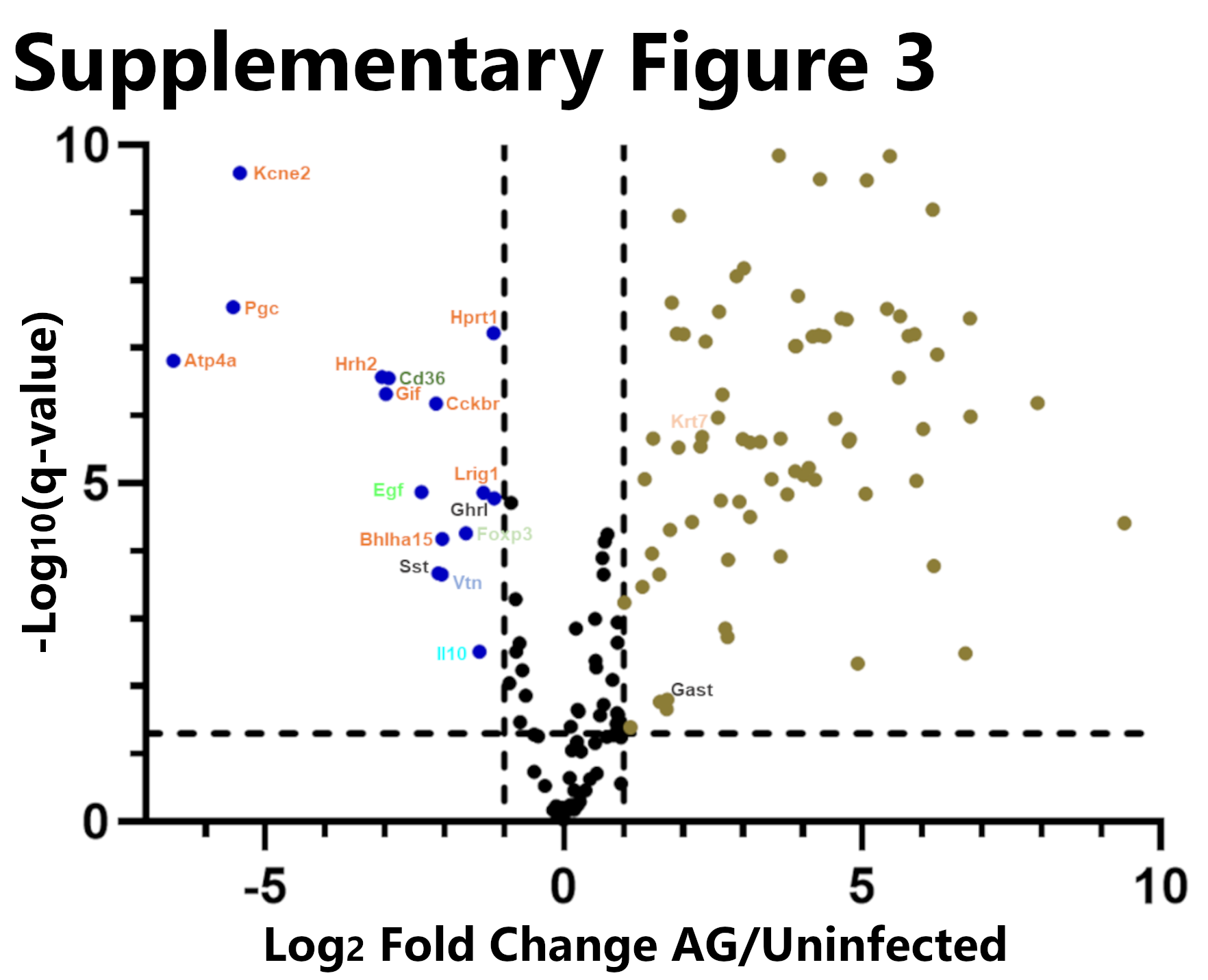

Supplement: Fig. S3 — Gastric transcriptomes of H. pylori-infected animals with atrophic gastritis compared to uninfected animals. [file msystems.01098-23-s0004.tif]

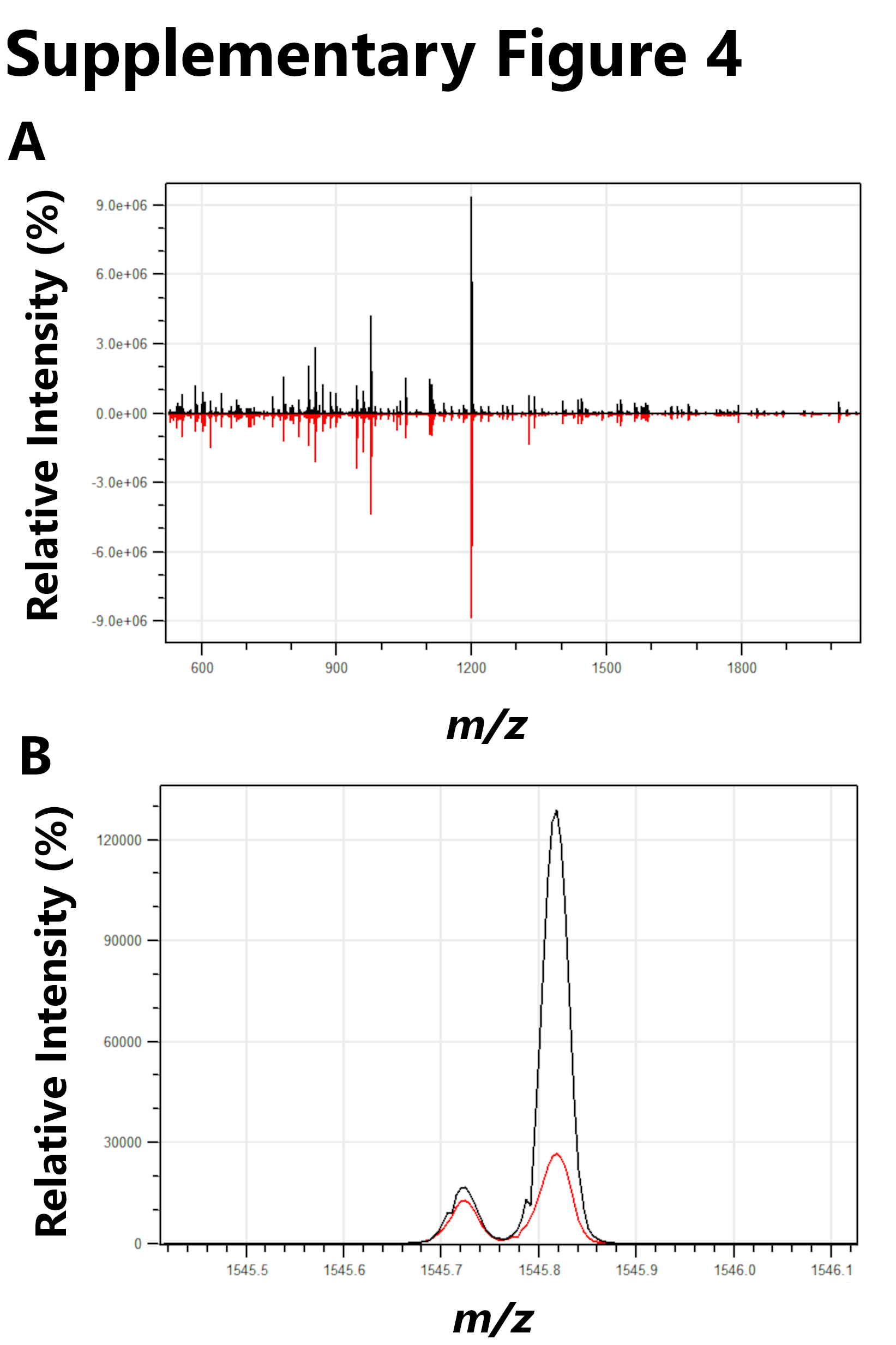

Supplement: Fig. S4 — Averaged spectra of gastric peptides from representative gerbil stomachs. [file msystems.01098-23-s0005.tif]

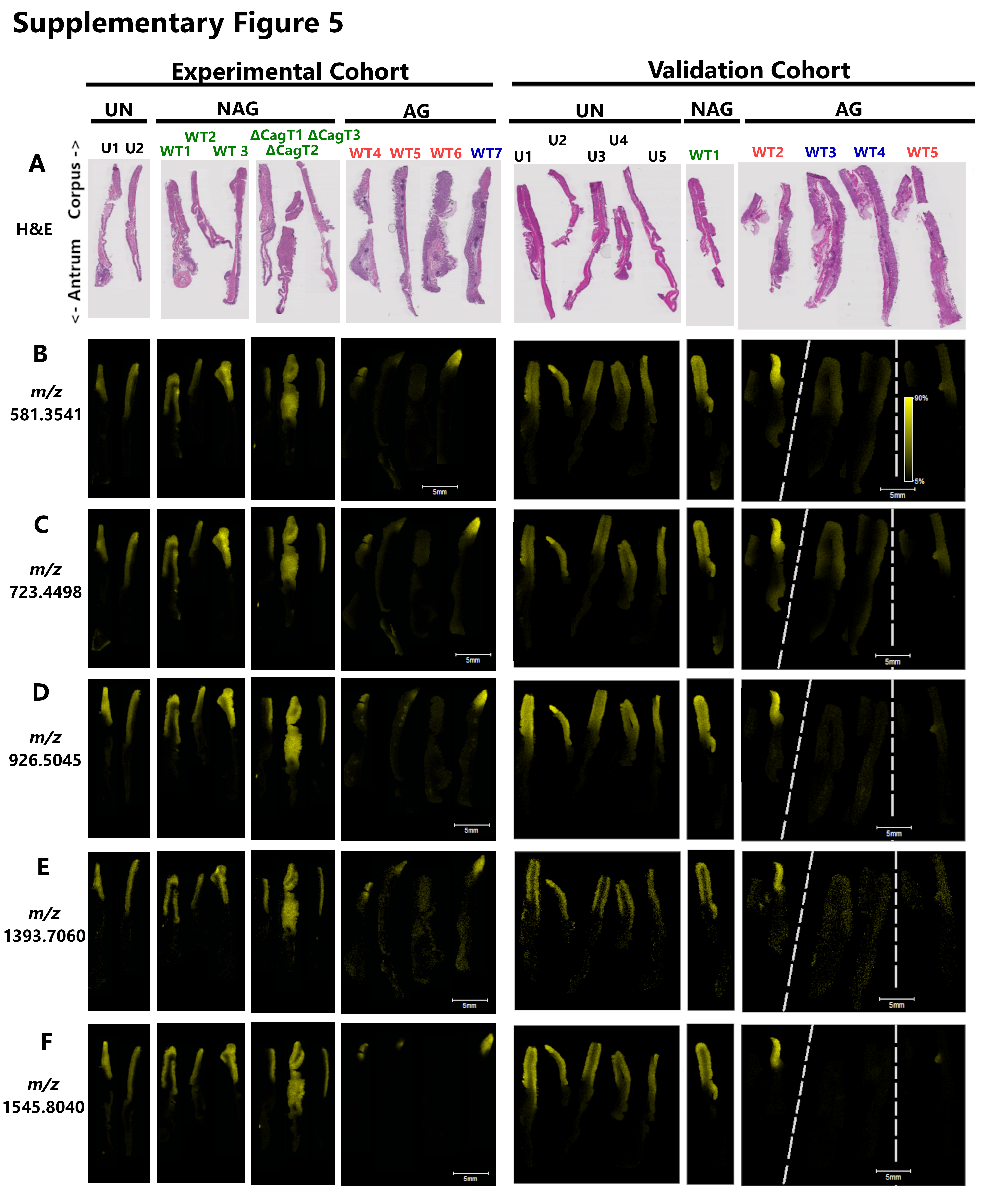

Supplement: Fig. S5 — IMS analysis depicting peptides localized to the corpus in uninfected tissues and decreased in abundance in infected stomachs with atrophic gastritis (monochrome images). [file msystems.01098-23-s0006.tif]

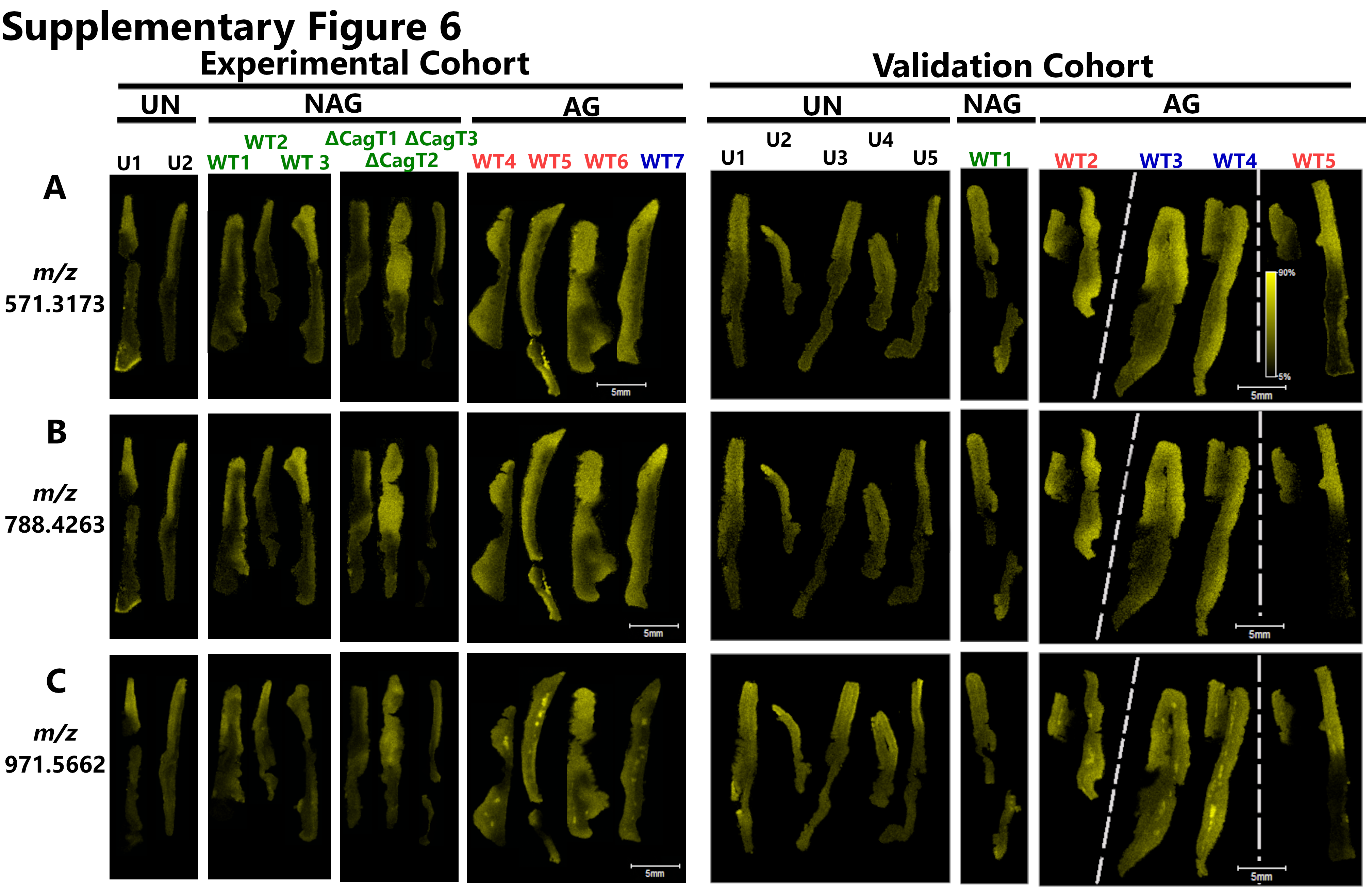

Supplement: Fig. S6 — IMS analysis depicting peptides localized preferentially to the corpus in uninfected animals and delocalized throughout the stomach in infected animals with atrophic gastritis (monochrome images). [file msystems.01098-23-s0007.tif]

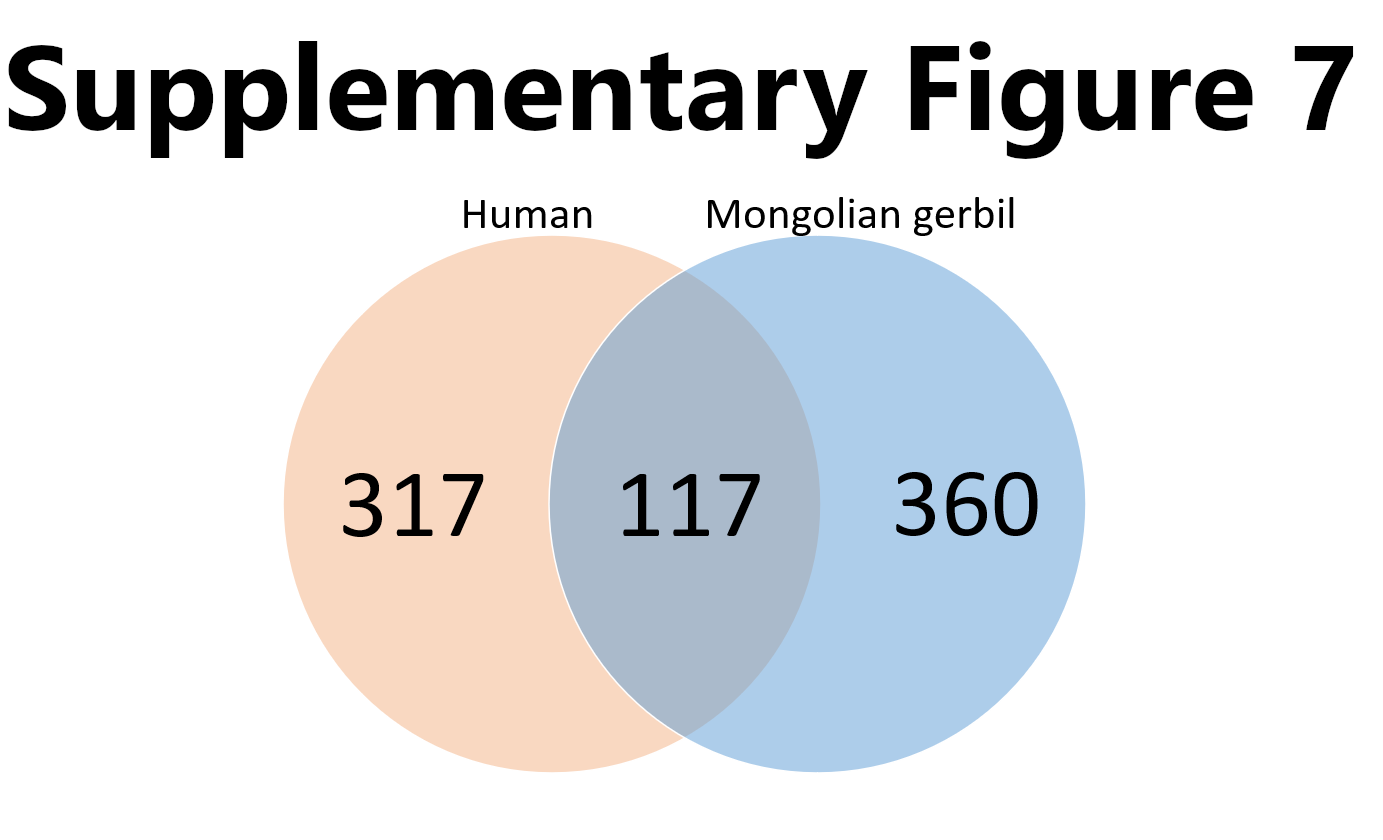

Supplement: Fig. S7 — Cross-species comparison of corpus-enriched proteins. [file msystems.01098-23-s0008.tif]
