# Supplementary material for: Remodeling of the gastric environment in Helicobacter pylori-induced atrophic gastritis
Source: mSystems. 2023 Dec 7;9(1):e01098-23. doi: 10.1128/msystems.01098-23 (PMC10805037; doi:10.1128/msystems.01098-23)
Supplement: Supplemental figure legends — Legends for Figures S1 to S7. [file msystems.01098-23-s0002.pdf]

Supplemental Figure Legends

Supplementary Figure S1: Description of Mongolian gerbil cohorts. Gastric tissues from three cohorts of Mongolian gerbils (both male and female) were analyzed by NanoString (Transcriptional Cohort) or by IMS and LC-MS/MS (Proteomic Experimental Cohort and Proteomic Validation Cohort). The Transcriptional Cohort was on regular rodent chow (Purina 5L0D) and infected with *H. pylori* 7.13 or mock-infected with Brucella broth alone. These animals were euthanized at 12- or 16-weeks post-infection. The Experimental Cohort was infected with *H. pylori* strain 7.13 or a Δ*cagT* mutant, or mock-infected. The Validation Cohort utilized archived tissues from a study in which gerbils were infected with *H. pylori* strain 7.13 or remained uninfected (no Brucella broth gavage). Both of the cohorts analyzed by proteomics received an AIN-93M diet beginning one week prior to infection and continued throughout the subsequent 12 weeks.

Supplementary Figure S2: Loss of Cag T4SS activity in Δ*cagT* mutants. Cag T4SS activity was assessed with an AGS reporter cell assay in which a luciferase gene was controlled by an NFκB-regulated promoter (1). A Δ*cagU* mutant (defective in T4SS activity) was tested as a negative control (2). As expected, the WT parental strain 7.13 exhibited Cag T4SS activity when co-cultured with AGS gastric epithelial cells (based on analysis of NFκB activation), whereas the Δ*cagT* isogenic mutant strains were inactive in this assay (p-value <0.0001).

Supplementary Figure S3: Gastric transcriptomes of *H. pylori*-infected animals with atrophic gastritis (AG) compared to uninfected animals. Transcriptional profiling of gastric

tissues was done using a custom-designed NanoString panel. Labels indicate selected genes that were differentially abundant. There were no statistically significant differences when comparing the transcriptional profiles of infected stomachs with non-atrophic gastritis to stomachs of uninfected animals (data not shown).

Supplementary Figure S4: Averaged spectra of peptides detected by IMS analysis of six representative gerbil stomachs. (A) Average mass spectra over whole sections of gastric tissue for two uninfected stomachs (shown in black) and four *H. pylori*-infected stomachs with atrophic gastritis (shown in red) from the Experimental cohort. Overall, the signal intensity and quality were similar in all tissues. (B) Zoomed-in spectra of the region around *m/z* 1545.8040 (identified as potassium-transporting ATPase subunit beta isoform X1), showing a peak that was more abundant in the uninfected tissues compared to the infected tissues (this peptide is also shown in Figure 5).

Supplementary Figure S5: Monochrome representation of main manuscript Figure 5, depicting peptides that are localized to the corpus in uninfected tissues and less abundant in *H. pylori*-infected stomachs with atrophic gastritis.

Supplementary Figure S6: Monochrome representation of main manuscript Figure 6, depicting peptides that localize preferentially to the corpus in uninfected animals and infected animals with non-atrophic gastritis but are delocalized throughout the stomach in infected animals with atrophic gastritis.

Supplementary Figure S7: Cross-species overlap of corpus-enriched proteins. About one-fourth of the corpus-enriched proteins detected in uninfected Mongolian gerbil tissue were also considered corpus-enriched in a previous proteomic study of histologically normal human tissue (3). A list of shared and unique corpus-specific proteins can be found in Supplementary Table S10.

1. Barrozo RM, Cooke CL, Hansen LM, Lam AM, Gaddy JA, Johnson EM, Cariaga TA, Suarez G, Peek RM, Jr., Cover TL, Solnick JV. 2013. Functional plasticity in the type IV secretion system of Helicobacter pylori. PLoS Pathog 9:e1003189.
2. Lin AS, McClain MS, Beckett AC, Caston RR, Harvey ML, Dixon B, Campbell AM, Shuman JHB, Sawhney N, Delgado AG, Loh JT, Piazuelo MB, Algood HMS, Cover TL. 2020. Temporal Control of the Helicobacter pylori Cag Type IV Secretion System in a Mongolian Gerbil Model of Gastric Carcinogenesis. mBio 11.
3. Ni X, Tan Z, Ding C, Zhang C, Song L, Yang S, Liu M, Jia R, Zhao C, Song L, Liu W, Zhou Q, Gong T, Li X, Tai Y, Zhu W, Shi T, Wang Y, Xu J, Zhen B, Qin J. 2019. A region-resolved mucosa proteome of the human stomach. Nat Commun 10:39.
