# Supplementary material for: Remodeling of the gastric environment in Helicobacter pylori-induced atrophic gastritis
Source: mSystems. 2023 Dec 7;9(1):e01098-23. doi: 10.1128/msystems.01098-23 (PMC10805037; doi:10.1128/msystems.01098-23)
Supplement: Additional experimental details — Supplemental methods. [file msystems.01098-23-s0009.pdf]

METHODS

H. pylori culture methods

H. pylori strain 7.13 is a derivative of the human clinical isolate B128 (1) that has been adapted to the Mongolian gerbil model. Rodent-adapted 7.13 has an intact *cag* pathogenicity island that encodes components of a functional Cag T4SS and the CagA oncoprotein, and it can cause gastric cancer in the Mongolian gerbil model. Wild-type 7.13 and a Δ*cagT* mutant strain (described below) were grown at 37°C on trypticase soy agar (TSA) plates containing 5% sheep blood in room air containing 5% supplemental CO_2. Prior to animal infection, strains were grown in Brucella broth containing 10% fetal bovine serum overnight in a shaking incubator (180 RPM) under the same conditions.

Generation of the Δ*cagT* mutant strain

To generate a Δ*cagT* mutant strain derived from the gerbil-adapted wild-type strain 7.13, we designed a plasmid containing a chloramphenicol acetyltransferase gene flanked by 500 base pairs of *H. pylori* genomic sequences on either side of *cagT* (from the *cagU* and *cagS* genes, as well as the intergenic region between *cagT* and *cagS)*. This plasmid does not replicate in *H. pylori*. After the plasmid was transformed into strain 7.13, transformants were selected by growth on sodium bisulfite-free Brucella agar plates supplemented with 2.5% fetal bovine serum and 5 ug/mL chloramphenicol. Transformants were passaged an additional time on chloramphenicol-containing plates. PCR analysis confirmed the deletion of *cagT* in the transformants, and Western blotting with an anti-CagT antiserum confirmed that CagT was not produced in the Δ*cagT* mutant strain (designated strain 7.13_5.2T). As expected, the wild-type parental strain exhibited Cag T4SS activity when co-cultured with AGS gastric epithelial cells (based on analysis of NF-kB activation), whereas the Δ*cagT* mutant strains were inactive in this assay (Supplementary Figure 2) (2). The Δ*cagT* mutant retained the capacity to colonize gerbil stomachs at rates and densities similar to the WT strain.

Mongolian gerbil infections

Male and female Mongolian gerbils (35 to 50 g weight) were obtained from Charles River Laboratories. One day prior to infection, gerbils were fasted overnight. The next day (referred to as day 0), the animals were orally gavaged with a 500 µL suspension (1×10^9 CFU/mL in Brucella broth) of *H. pylori* strain 7.13 or a pool of five Δ*cagT* mutant single colonies; the oral gavage was repeated on day 2. In parallel, uninfected control animals received unmanipulated Brucella broth by gavage (the transcriptionally analyzed cohort and the proteomic experimental cohort) or did not undergo mock-infection (the proteomic validation cohort). There were no notable differences in results when comparing uninfected animals that underwent mock infection with those that did not.

Description of Mongolian gerbil cohorts

The transcriptionally analyzed gerbil cohort received regular rodent chow (Purina 5L0D) and were infected with *H. pylori* 7.13 or mock-infected with Brucella broth alone. The animals were euthanized at 12- or 16-weeks post-infection. RNA isolated from these stomachs (an equal mix of animals infected for 12 or 16 weeks) was analyzed by NanoString. An additional gerbil cohort (the "experimental cohort") received an AIN-93M rodent diet (Bio-Serv) diet for one week and were then infected with *H. pylori* 7.13, the Δ*cagT* knockout mutant, or mock-infected. These animals were euthanized at 12 weeks post-infection and their stomachs were analyzed by imaging mass spectrometry (IMS) and LC-MS/MS. To validate the IMS and LC-MS results, we analyzed archived gastric tissues from an independent cohort (the "validation cohort," described and analyzed in a previous study (3)) that also received AIN-93M diet. These animals were infected with *H. pylori* 7.13 for 12 weeks; the uninfected control animals in this group did not undergo oral gavage with Brucella broth.

In the transcriptionally analyzed cohort, atrophic gastritis with dysplasia and/or adenocarcinoma developed in about 18% of animals infected for 12-16 weeks (21% at 16 weeks and 15% at 12 weeks). The proteomic experimental cohort was fed an AIN-93M diet (39 ppm iron compared to 250 ppm iron in the Purina 5L0D diet) to induce higher disease rates, as described in previous studies (3, 4). In the proteomic experimental cohort, atrophic gastritis developed in 70% of the animals infected with a wild-type strain, and none of the animals infected with the Δ*cagT* mutant strain; the Δ*cagT* mutant-infected animals had inflammation scores and disease diagnoses similar to those of the animals infected with the wild-type strain that only developed non-atrophic gastritis.

Processing of gastric tissue

Gerbil stomachs were dissected to remove the nonglandular forestomach. The glandular region (including the corpus and antrum) was cut along the lesser curvature and laid flat for processing. Surgical dye was used to mark the corpus of one-third to half of the glandular stomach, which was then placed in a plastic cassette wrapped with aluminum foil, flash-frozen in dry ice, and stored at −70°C; this portion was subsequently used for IMS analysis. Another portion of the glandular stomach was fixed in 10% neutral buffered formalin and embedded in paraffin. The histology of the formalin-fixed paraffin-embedded (FFPE) tissues was analyzed as described below.

Histology

Strips of stomach containing both corpus and antrum were fixed in 10% neutral buffered formalin for about 24 hours. Then, each tissue was embedded in paraffin, sectioned onto a slide, and stained with hematoxylin and eosin (H&E). A gastrointestinal pathologist examined the H&E-stained sections of gastric tissues in a blinded fashion.

Inflammation scores (0, 1, 2, and 3, corresponding to absent, mild, moderate, or marked inflammation, respectively) were assigned to evaluate both acute (neutrophils/polymorphonuclear leukocytes) and chronic (mononuclear leukocytes) inflammation in the antrum and in the corpus, resulting in a total inflammation score of 0-12. Histologic analysis included an evaluation of the presence of atrophic gastritis (reported as a “corpus atrophy” score on a scale of 0, 1, 2, or 3, corresponding to 0%, 1-33%, 24-66%, or >67% loss of parietal and chief cells), dysplasia, or gastric adenocarcinoma (5).

RNA extraction and purification

RNA was extracted from whole strips of stomach tissue, including both the corpus and antrum, using the acid-guanidinium-phenol (Trizol)-chloroform method. In brief, samples were homogenized in 1 mL TRIzol reagent (Invitrogen; Waltham, Massachusetts, USA). Next, isolated RNA underwent DNase I treatment according to the manufacturer’s protocol (NEB). Next, the GeneJet RNA Cleanup and Concentration Micro Kit was used according to the manufacturer’s protocol. These protocols resulted in an end volume of 60-100 µL of purified RNA, which was stored at -70ºC until subsequent analysis.

Design of a Mongolian gerbil NanoString panel

We selected 148 genes of interest grouped into 27 functional categories for inclusion in the custom-designed Mongolian gerbil NanoString panel. Genes were exclusively assigned to one category (e.g., a gene would not be considered both a proinflammatory cytokine and a chemokine). Genes on the panel included markers for immune system activation and markers for specific gastric cell types; specific cell type markers were chosen based on genes commonly reported as cell type markers in the literature (for example, *Atp4a, Gif, Hrh2,* and *Kcne2* for gastric parietal cells, and *Pgc* and *Bhlha15* for chief cells). Other markers of gastric function included genes for gastric hormones, such as *Sst* (somatostatin, produced by antral D cells), *Gast* (gastrin, produced by antral G cells), and *Ghrl* (Ghrelin, made by P/D1 endocrine cells in the oxyntic glands). Five housekeeping genes were also included. To identify the corresponding gene in the Mongolian gerbil, a BLASTn of each corresponding mouse ortholog gene’s sequence against the Mongolian gerbil genome was completed (6, 7). Next, the Mongolian gerbil protein coding sequence for each gene of interest underwent a protein-to-protein BLASTp search within the mouse proteome to determine amino acid sequence relatedness and increase confidence in the quality of the match. These Mongolian gerbil gene sequences were then used for customized panel design and manufacture (NanoString; Seattle, Washington, USA). The targets for the manufactured probes are listed in Supplementary Table 3.

Analysis of NanoString data

RNA quality was assessed using a Qubit machine, and then 100 ng of RNA was hybridized to the custom Mongolian gerbil NanoString panel according to NanoString's published protocol, using resources in the Vanderbilt Technologies for Advanced Genomics (VANTAGE) core. Hybridized samples were processed on the nCounter prep station and scanned on the NanoString digital analyzer to generate RCC data files.

RCC data files were imported, processed, analyzed, and exported according to the nSolver 4.0 Analysis Software User Manual. Data were checked for quality control using the Imaging QC, Binding Density QC, Positive Control Linearity QC, and Positive Control Limit of Detection QC metrics within the software. Data were normalized using the geometric mean of positive control counts and housekeeping gene counts. NanoString Advanced Analysis software was used to create a heat map, and to compare gene counts from groups of animal tissues selected for comparison.

All pairwise ratios (infected animals with atrophic gastritis compared to infected animals with non-atrophic gastritis; infected animals with atrophic gastritis compared to uninfected animals; and infected animals with non-atrophic gastritis compared to uninfected animals) were analyzed to generate a $\log_2$ fold change and a t-test was used to generate a p-value. Benjamini-Hochberg multiple test correction was used to generate a corrected p-value (q-value). The pairwise ratio data were visualized using GraphPad.

Slide Preparation for Mass Spectrometry Analysis

Stomachs were selected for analysis by IMS and LC-MS/MS based on inflammation score and disease state. Gastric inflammation was not detected in any of the uninfected animals, so uninfected stomachs were selected for analysis at random.

Fresh frozen gerbil stomach tissues were sectioned into 12 µm thick sections using a cryostat (Leica CM 3050S, Leica Biosystems, Nußloch, Germany). Not all tissue samples from a cohort could fit on a single slide, so tissues from animals within a cohort were distributed onto two slides. Each slide contained infected and uninfected tissues. Specifically, the stomachs were affixed to a cryostat chuck with optimal cutting temperature (OCT) polymer, and 12 µm sections were cut and thaw-mounted onto indium-tin oxide (ITO)-coated glass slides (Delta Technologies, Ltd.). Serial sections were obtained for tryptic digest imaging and H&E staining. Slides were placed in a slide mailer, flushed with nitrogen gas, sealed and stored at -80°C until analysis.

Slide Processing for Mass Spectrometry Analysis

Gastric samples for analysis by MALDI and LC-MS/MS were washed to remove salts and lipids as previously described(8). Specifically, slides were dipped in and out of Coplin jars as follows: 70% ethanol for 30 seconds; 100% ethanol for 30 seconds; Carnoy's solution (6:3:1, ethanol:chloroform:acetic acid) for 2 minutes; 100% ethanol for

30 seconds; 40% ethanol for 30 seconds; 100% ethanol for 30 seconds; following by drying in the hood for 10 minutes. After washing, a total of 3.2 ng/mm2 (final) trypsin (Pierce™ Trypsin Protease, MS Grade) was applied to the entire tissue section via an HTX M3 TM sprayer as previously described(8). Next, tissues were digested overnight (roughly 16 hours) in a humidity oven (Espec) at 37°C, 100% relative humidity.

Imaging Mass Spectrometry (MALDI) analysis of peptides

For matrix application, 5 mg/mL α-cyano-4-hydroxycinnamic acid in 90% acetonitrile, 0.1% TFA was applied using an HTX M3 TM sprayer set to 8 passes, 85°C nozzle, 700 mm/min, 45°C stage, and 2 mm track spacing. Peptide images were obtained on a 15 Tesla Bruker SolariX FT-ICR mass spectrometer in positive ion mode. Tryptic peptide images from m/z 500 – 3500 were acquired using a time-domain file size of 512K, resulting in an ICR transient length of 0.5767 sec and a resolving power of ~65,000 at *m/z* 1200 with 75-micron lateral resolution, 100 shots per pixel, medium laser focus, and no random walk. Uninfected and infected tissues from the same cohort were always sectioned, prepared, and analyzed together.

Analysis of MALDI data

We used flexImaging software (Bruker Daltonics) to manually analyze each peak in the mass spectrum and thereby identify signals of interest; no automated algorithms or supplemental analysis programs were used. Total ion current (TIC) normalization was used to normalize imaging patterns. We first analyzed the data to detect imaging peptides that were enriched in the corpus (compared to the antrum) in tissues from uninfected animals. We then did further analysis of these corpus-specific peaks to identify those that were decreased in abundance or delocalized in tissues from infected animals with atrophic gastritis compared to tissues from uninfected animals. This analysis resulted in identification of nine corpus-specific peptides in uninfected animals that were decreased in abundance in animals with atrophic gastritis and four corpus-specific peptides in uninfected animals that were delocalized in animals with atrophic gastritis. Other patterns that were reproducibly detected in the two biologically independent cohorts include increased abundance of one peptide in the antrum of animals with atrophic gastritis (*m/z* 1933.9280), and increased abundance of one peptide in the corpus of animals with atrophic gastritis (*m/z* 958.5696), compared to uninfected animals (images not shown).

The grouping of tissues shown in figures is not identical to the organization of the original slides, which were laid out with the tissues mixed together (not separated by infection or disease status) to minimize bias. Tissues from the experimental and validation cohorts (described further in the "Description of Mongolian gerbil cohorts" section above) were analyzed independently of each other. After generation of a list of discriminatory peaks within one cohort, the lists were cross-compared to tissues from the other

experiment, generating a list of imaging peaks that were reproducibly altered in two independent animal cohorts.

LC-MS/MS Sample Preparation

Following trypsin digestion of tissue sections overnight (described in the "Slide Processing for Mass Spectrometry Analysis" section above), peptides were microextracted by pipetting 2 µL 40% acetonitrile, 0.1% formic acid in HPLC water up and down ten times in either the corpus or antrum region of the stomach. The extract was placed in an Eppendorf LoBind® tube; when there was ample area of the corpus or antrum, two individual microextractions per area were performed and pooled together in the Eppendorf LoBind® tube. Extracts were dried in a Speedvac with no heat and stored at -80°C until ready for LC-MS/MS analysis. Samples were reconstituted in 10 µl 0.2% formic acid and 0.8 µl were injected onto 100 um x 25 cm self-packed reversed phase column coupled to a nano-electrospray source such that eluting peptides would be directly ionized into the inlet of an Exploris 480 (Thermo-Fisher) mass spectrometer. Peptides were resolved using an RSLC-nano (Thermo-Fisher) via an aqueous to organic gradient with a total run time of 90 minutes and data acquired via data-dependent acquisition where the top 20 signals from each full MS1 scan were selected for tandem fragmentation (MS/MS). MS1 scan windows were from *m/z* 375 – 1600A minimal signal threshold of 10,000 was used, including only charge states 2 – 5, a maximum inject time of 100 ms, and a dynamic exclusion window of 20 seconds.

LC-MS/MS Data Analysis

LC-MS/MS results were analyzed using FragPipe (software for FragPipe, a complete proteomics pipeline with the MSFragger search engine at heart, can be found at nesvilab.org). For identifications and MS1 quantitation via IonQuant (9), results were searched against a Mongolian gerbil protein database downloaded from NCBI-RefSeq appended to an *H. pylori* protein database, with default parameters for LFQ-MBR searches performed using MSFragger (10). Pairwise comparisons between groups (uninfected, wild-type infected with non-atrophic gastritis, wild-type infected with atrophic gastritis, and *ΔcagT* mutant strain-infected with non-atrophic gastritis) and between gastric regions (antrum and corpus) were performed using MSStats (11). Search results filtered to a 1% false discovery rate using Percolator (12) were also loaded into Scaffold (Scaffold | Proteome Software, Inc., Portland, OR) for visualization and comparisons.

To determine which peptides detected by LC-MS/MS were corpus-specific and lost in atrophic gastritis, MSStats results were analyzed in several steps. First, proteins from uninfected stomachs with a statistically significant Log_2 Fold Change of one of more when comparing uninfected corpora to uninfected antra were identified (to determine which proteins were corpus-specific). Next, peptides with a Log_2 Fold Change of negative one or less when comparing the corpora of infected animals with atrophic gastritis to the

corpora of uninfected animals were removed from the list (to identify proteins that were lost in atrophic gastritis).

To determine which peptides detected by LC-MS/MS were corpus-specific and delocalized throughout the stomach in the context of atrophic gastritis, MSStats data comparisons were again used. First, proteins in antra from animals with atrophic gastritis were compared to those in uninfected antra, and any peptides with a statistically significant Log_2 Fold Change of one or more were selected (to identify peptides that are different in the antrum in the setting of atrophic gastritis). Next, only hits that are corpus-specific in uninfected animals were selected. This was done by removing hits with a Log_2 Fold Change of less than one when comparing uninfected corpora to uninfected antra. Finally, hits that decreased in the corpus in the setting of atrophic gastritis were removed by removing peptides with a Log_2 Fold Change of less than negative one when comparing corpora with atrophic gastritis to uninfected corpora.

Matching IMS images to LC-MS/MS Peptide Identifications

Using Scaffold, a list of proteins with 4 or more peptide hits from the LC-MS/MS analysis was exported. To compare LC-MS/MS data to IMS data with the goal of matching probable identifications for imaged peptides of interest, we calculated a parts per million (ppm) difference as follows:

$$\frac{(\text{IMS peptide mass} - \text{detected LC-MS/MS peptide mass}) * 1{,}000{,}000}{\text{Detected LC-MS/MS peptide mass}}$$

A ppm difference cutoff of 10 was used to narrow down the list of LC-MS/MS potential identifications to each biologically interesting IMS mass pattern, resulting in ~1-10 hits per IMS mass. Next, the relative quantitative peptide abundances from LC-MS/MS were compared to the IMS mass patterns, and if the pattern did not match, the LC-MS/MS peptide was dropped from the matching process; this resulted in 1-6 LC-MS/MS peptide matches for each IMS mass. Then, additional LC-MS/MS peptide characteristics, such as "Best Peptide Identification Probability" and "Total Ion Current (TIC)" were considered as evidence that one LC-MS/MS peptide was a better match than others; for example, LC-MS/MS peptides with a logarithmically higher total TIC were considered more likely matches. Finally, available literature was searched to provide additional support for potential LC-MS/MS peptide matches to IMS masses of interest. This information is compiled in Supplementary Table 4.

Comparison of Mongolian gerbil and human proteomic data

A previous proteomic analysis of human gastric tissue reported the identification of proteins that were differentially abundant in seven regions of the stomach(13). To compare results of this human study with proteomic analyses of gerbil corpus and antrum in the current study, we considered the "Greater Curvature" and "Lesser Curvature" regions from the human study to approximate the corpus, and the "Angular Incisure,"

"Antrum," and "Pylorus" regions from the human study to approximate the antrum. The current Mongolian gerbil study analyzed uninfected tissues and tissues with *H. pylori*-induced atrophic gastritis, while the human study analyzed "apparently normal" tissues as well as tumor and tumor-adjacent tissue from patients with diffuse-type gastric cancer. Supplemental Figure 7 shows the overlap in corpus-specific proteins detected in uninfected Mongolian gerbil tissues in the current study and normal human gastric tissues. A list of these proteins, along with corpus-specific proteins detected uniquely in human or gerbil gastric tissues, is shown in Supplemental Table 10.

Pathway Analysis

Starting with the three lists compiled in LC-MS/MS analysis of differential abundance described above (corpus-specific proteins in uninfected animals; corpus-specific proteins decreased or lost in atrophic gastritis; and corpus-specific proteins delocalized in atrophic gastritis), we converted Mongolian gerbil protein accession numbers from the LC-MS/MS data to mouse gene names. A protein-to-protein BLAST was conducted for each Mongolian gerbil protein identified by LC-MS/MS against the mouse proteome, which was then used to find the official gene symbol in the mouse. Mouse gene names were used due to relatively poor annotation of the Mongolian gerbil genome. These official gene symbols were entered into the Database for Annotation, Visualization, and Integrated Discovery (DAVID) as a gene list with *Mus musculus* as the species. The resulting KEGG pathways were used for further enrichment analysis. Enrichment scores are used to rank biological significance. Enrichment scores are calculated using the geometric mean (in $-\log_{10}$(p-value) scale) of each term member within the annotation group.

1. Noto JM, Chopra A, Loh JT, Romero-Gallo J, Piazuelo MB, Watson M, Leary S, Beckett AC, Wilson KT, Cover TL, Mallal S, Israel DA, Peek RM. 2018. Pan-genomic analyses identify key Helicobacter pylori pathogenic loci modified by carcinogenic host microenvironments. Gut 67:1793-1804.
2. Barrozo RM, Cooke CL, Hansen LM, Lam AM, Gaddy JA, Johnson EM, Cariaga TA, Suarez G, Peek RM, Jr., Cover TL, Solnick JV. 2013. Functional plasticity in the type IV secretion system of Helicobacter pylori. PLoS Pathog 9:e1003189.
3. Lin AS, Shuman JHB, Kotnala A, Shaw JA, Beckett AC, Harvey JL, Tuck M, Dixon B, Reyzer ML, Algood HMS, Schey KL, Piazuelo MB, Cover TL. 2021. Loss of Corpus-Specific Lipids in Helicobacter pylori-Induced Atrophic Gastritis. mSphere 6:e0082621.
4. Beckett AC, Piazuelo MB, Noto JM, Peek RM, Jr., Washington MK, Algood HM, Cover TL. 2016. Dietary Composition Influences Incidence of Helicobacter pylori-Induced Iron Deficiency Anemia and Gastric Ulceration. Infect Immun 84:3338-3349.
5. Piazuelo MB, Correa P. 2013. Gastric cancer: Overview. Colomb Med (Cali) 44:192-201.
6. Zorio DAR, Monsma S, Sanes DH, Golding NL, Rubel EW, Wang Y. 2019. De novo sequencing and initial annotation of the Mongolian gerbil (Meriones unguiculatus) genome. Genomics 111:441-449.

7. Cheng SF, Fu Y, Zhang YL, Xian WF, Wang HL, Grothe B, Liu X, Xu X, Klug A, McCullagh EA. 2019. Enhancement of de novo sequencing, assembly and annotation of the Mongolian gerbil genome with transcriptome sequencing and assembly from several different tissues. Bmc Genomics 20.

8. Judd AM, Gutierrez DB, Moore JL, Patterson NH, Yang J, Romer CE, Norris JL, Caprioli RM. 2019. A recommended and verified procedure for in situ tryptic digestion of formalin-fixed paraffin-embedded tissues for analysis by matrix-assisted laser desorption/ionization imaging mass spectrometry. Journal of Mass Spectrometry 54:716-727.

9. Yu F, Haynes SE, Nesvizhskii AI. 2021. IonQuant Enables Accurate and Sensitive Label-Free Quantification With FDR-Controlled Match-Between-Runs. Molecular & Cellular Proteomics 20:100077.

10. Kong AT, Leprevost FV, Avtonomov DM, Mellacheruvu D, Nesvizhskii AI. 2017. MSFragger: ultrafast and comprehensive peptide identification in mass spectrometry–based proteomics. Nature Methods 14:513-520.

11. Choi M, Chang CY, Clough T, Broudy D, Killeen T, MacLean B, Vitek O. 2014. MSstats: an R package for statistical analysis of quantitative mass spectrometry-based proteomic experiments. Bioinformatics 30:2524-6.

12. The M, MacCoss MJ, Noble WS, Kall L. 2016. Fast and Accurate Protein False Discovery Rates on Large-Scale Proteomics Data Sets with Percolator 3.0. J Am Soc Mass Spectrom 27:1719-1727.

13. Ni X, Tan Z, Ding C, Zhang C, Song L, Yang S, Liu M, Jia R, Zhao C, Song L, Liu W, Zhou Q, Gong T, Li X, Tai Y, Zhu W, Shi T, Wang Y, Xu J, Zhen B, Qin J. 2019. A region-resolved mucosa proteome of the human stomach. Nat Commun 10:39.
